# Preclinical model of Echinacea-functionalized silk fibroin–chitosan transparent membranes with enhanced hair follicle regeneration and antibacterial properties for wound healing

**DOI:** 10.3389/fbioe.2026.1806381

**Published:** 2026-07-06

**Authors:** Zhili Wei, Lunan Yang, Sining Feng, Bowen Zheng, Xingliang Xiong, Yiming Zhao

**Affiliations:** 1 Department of Clinical Laboratory Center, Beijing Children’s Hospital, Capital Medical University, National Center for Children’s Health, Beijing, China; 2 Peking University People’s Hospital, Beijing, China; 3 Department of Plastic and Aesthetic Surgery, Nanfang Hospital, Southern Medical University, Guangzhou, China; 4 College of Medical Informatics, Chongqing Medical University, Chongqing, China

**Keywords:** echinacea, hair follicle regeneration, hemostasis, sacless wound healing, silk fibroin, transparent dressing

## Abstract

**Introduction:**

Skin serves as a crucial barrier that separates the human body from the external environment. Its complex structure can make it susceptible to microbial infection and bleeding, leading to poor wound healing. Therefore, it is of important clinical significance to develop a wound dressing that can form scarless tissues with hair follicles, inhibit bacterial infection, and promote hemostasis.

**Methods:**

In this study, we constructed composite dressings based on Echinacea-loaded silk fibroin/chitosan (ESC) that had excellent mechanical properties and air permeability for the promotion of hair follicle regeneration and scarless wound healing. Additionally, the transparency of ESC facilitated observation of the wound healing process.

**Results:**

ESC exhibits superior biocompatibility that effectively alleviates inflammatory reactions in wounds. In vitro assays showed that ESC has excellent tissue adhesion and hemostatic abilities. In vivo wound healing assays comparing SF-CS and commercial Tegaderm™ Film groups showed that ESC could accelerate wound angiogenesis, collagen deposition, keratinocyte proliferation, macrophage M1-M2 transition, and inhibit inflammation, indicating effective promotion of scarless skin regeneration.

**Discussion:**

These transparent dressings based on natural materials show great potential for scarless skin regeneration and treating acute injury hemostasis.

## 1 Introduction

Skin, the largest organ of the human body, plays an important role in preventing pathogen invasion from the external environment and protecting internal tissues (Zhang et al., 2019; Zheng et al., 2020). However, when skin is traumatized, it is susceptible to exogenous biological infections and chronic ulcers, leading to the destruction of deep muscles and making wound healing difficult (Castaño et al., 2018). Therefore, regeneration of the entire skin layer, composed of epi-dermis, dermis, and skin appendages, is challenging (Kang et al., 2023; Kang et al., 2021). Bacterial infection often occurs following skin trauma, which seriously affects the wound-healing process and causes more severe damage to the skin (Cheng et al., 2024). Therefore, wound dressings that can pro-mote skin wound healing, effectively inhibit bacterial infection, and absorb wound exudates are of great significance for clinical treatment.

Dressings in the form of hydrogels and nano-scaffolds have been developed. Although antibacterial hydrogels developed by Zhao and others can effectively promote wound healing (Jia et al., 2023; Zhao et al., 2023), they are limited by their material characteristics. Usually, hydrogel dressings struggle to allow sufficient oxygen exchange between the wound and the outside world, and they do not allow observation of wound changes in re-al-time. Although the scaffold material prepared by Xie using silk fibroin (SF) (Xie et al., 2022), sodium alginate, and urea has good transparency and air permeability, it lacks hydrophilicity and struggles to aborb wound exudates (Zhang et al., 2017). This leads to a large number of nutrients oozing out, creating a suitable environment for bacterial growth and making the wound more susceptible to infection. Sharda et al. (Gupta et al., 2022) prepared nanofiber patches by fusing ions with SF via electrostatic spinning technology, which conferred antimicrobial and wound-healing properties. Li et al. (Li et al., 2022) spray coated nano microspheres onto SF-based film to facilitate wound exudate absorption and promote wound healing. Both studies make use of the capacity of membrane dressings to absorb wound exudates and increase drug-carrying capacity. Therefore, preparing dressings that protect wounds from microbial infection, absorb wound exudates, possess good air permeability, and promote skin regeneration without causing scarring remains a major goal of materials science.

SF is a natural protein extracted from silkworm cocoons that has good biocompatibility, low immunogenicity, and high biodegradability. At both physiological and molecular levels, SF is involved in four key stages of wound healing: inflammation (Chouhan and Mandal, 2020; Martin and Nunan, 2015) blood clotting, cell proliferation, and extracellular matrix remodeling (Ma et al., 2023; Ueno et al., 1999). Studies have shown that SF can reduce inflammation and promote blood vessel formation to accelerate wound healing (Yu et al., 2022). In addition, as a natural material, SF has lower inflammatory reactivity and better skin affinity than traditional wound materials (Speroni et al., 2002). In *in vivo* experiments, SF film used for wound treatment did not induce wound exudates (Ju et al., 2016), and its promotion of epithelialization in full-thickness wound models accelerated wound healing, resulting in better treatment of full-thickness skin injuries. In a study on diabetic wounds, as a carrier for loading insulin, SF significantly accelerated the healing of skin wounds in diabetic rats (Li et al., 2017; Yang et al., 2020). However, SF does not possess antibacterial activity, and the naturally brittle and easily breakable nature of pure SF film limits its use in wound healing. Therefore, further research is needed on how to better utilize the advantages of SF in wound healing.

Chitosan (CS), a natural polysaccharide polymer extracted via partial deacetylation of chitin, has good film-forming ability. Different ratios of CS can be used to con-struct scaffold materials with specific mechanical, permeability, transparency, moisture absorption, and moisturizing properties, thereby improving the physical and biological properties of loaded material systems. Therefore, CS is a candidate material for pre-paring three-dimensional scaffolds (Jayakumar et al., 2011). Although CS has potential as a load-bearing scaffold material, pure CS has high adhesion and low permeability. Therefore, in the present work, CS was mixed with SF to prepare SF-CS polymer. This improved the performance of the load-bearing scaffold, making it tough, elastic, and permeable, creating a better mechanical environment for wound repair (Moeini et al., 2020). In addition, as a positively charged alkaline polysaccharide, CS participates in various stages of wound healing. In the early stages, its unique antibacterial adhesive (Hou et al., 2022), and hemostatic functions promote the migration and infiltration of polymorphonuclear neutrophils and macrophages and induce the proliferation of dermal fibroblasts, thereby accelerating the formation of granulation tissue (Howling et al., 2001; Ueno et al., 1999). In the middle and later stages of wound healing, CS can effectively reduce scar tissue, promote microvascular regeneration, accelerate re-epithelialization, and thus promote skin wound healing.

Echinacea purpurea is a natural herbaceous plant containing substances such as caffeic acid (Xu et al., 2021), chicoric acid, and echinacoside (Weishaupt et al., 2020) that have been recognized by the World Health Organization as safe botanical preparations (Moghtaderi et al., 2021), which has been proven to possess immunomodulatory, anti-inflammatory, and antibacterial properties, and has been used in clinical practice for treating various respiratory symptoms caused by bacterial infections (Karsch-Völk et al., 2014), chronic arthritis (Jiang et al., 2014), cancer (Hosami et al., 2021), and trauma (Soon and Crawford, 2001). In the field of wound healing, the multiple active ingredients in Echinacea can improve skin hydration and accelerate wound healing (Ciganović et al., 2023). In addition, polyphenols in Echinacea neutralize free radicals and destructive reactive oxygen species, thereby reducing skin damage and reduce the inflammatory process in wound healing (Barrett, 2003). However, natural plant extracts are usually in the form of powder or extract liquid, which cannot be used directly as a wound dressing for wound healing; hence, it is necessary to further process Echinacea powder into wound dressing forms to meet clinical needs.

Herein, a new porous drug-loaded scaffold dressing was prepared using Echinacea-loaded SF-CS (ESC) for wound healing. The material has many characteristics in terms of physical properties; the SF-CS scaffold provides the dressing with high adhesion, high drug loading rate, high permeability and transparency, and high dry and wet mechanical properties; *in vitro* experiments showed that the ESC dressing, made of pure natural materials, has excellent biocompatibility and antibacterial properties, and no cytotoxicity; *in vivo* experiments showed that the dressing could effectively accelerate wound healing in a mouse wound model. Histological staining results showed that the dressing promoted angiogenesis, inhibited inflammation, and promoted collagen regeneration. It also accelerated the transition of macrophages from M1 to M2 and demonstrated good hemostatic ability. Overall, the results show that this completely natural wound dressing participates in four key stages of wound healing, thereby accelerating this process. The findings provide inspiration for new 3D scaffold materials in wound repair. The low cost and simple preparation process make this material ideal for scarless skin recovery with hair follicle regeneration.

## 2 Materials and methods

### 2.1 Materials

The cocoons of *Bombyx mori* were obtained from Southwest University. Chitosan was purchased from Shanghai Aladdin Co., Ltd. (Shanghai, China) Echinacea extract (ethanol extract) was purchased from Xi’an Xinlu Biotechnology Co. (Xi’an, China). This product is a finished biological product manufactured on a standardized production line and complies with Chinese national sampling inspection standards. LB broth medium and AGAR powder was purchased from Shanghai Sangon Co., LTD., Ltd. (Shanghai, China) High glucose medium was purchased from Gibco. Fetal bovine serum and double antibodies were purchased from Sijiqing Co., Ltd. (Hangzhou, China) CK10, Interleukin 6 (IL-6), and VEGF antibodies were purchased from Abcam and servicebio, respectively. Cell Counting Kit-8 (CCK8) and DPPH free radical scavenging capacity analysis kit were purchased from Beijing Solarbio Science and Technology Co., Ltd. (Beijing, China) Rhodamine phalloidin was purchased from Sigma. Other reagents used in this work are analytical-grade reagents. The Tegaderm™ Film is from the 3M technology company (Minnesota, United States). Deionized (DI) water (resistance: 18.25 MΩ cm^−1^) was used during the experiment.

### 2.2 Preparation of silk fibroin solution

**FIGURE 1:**
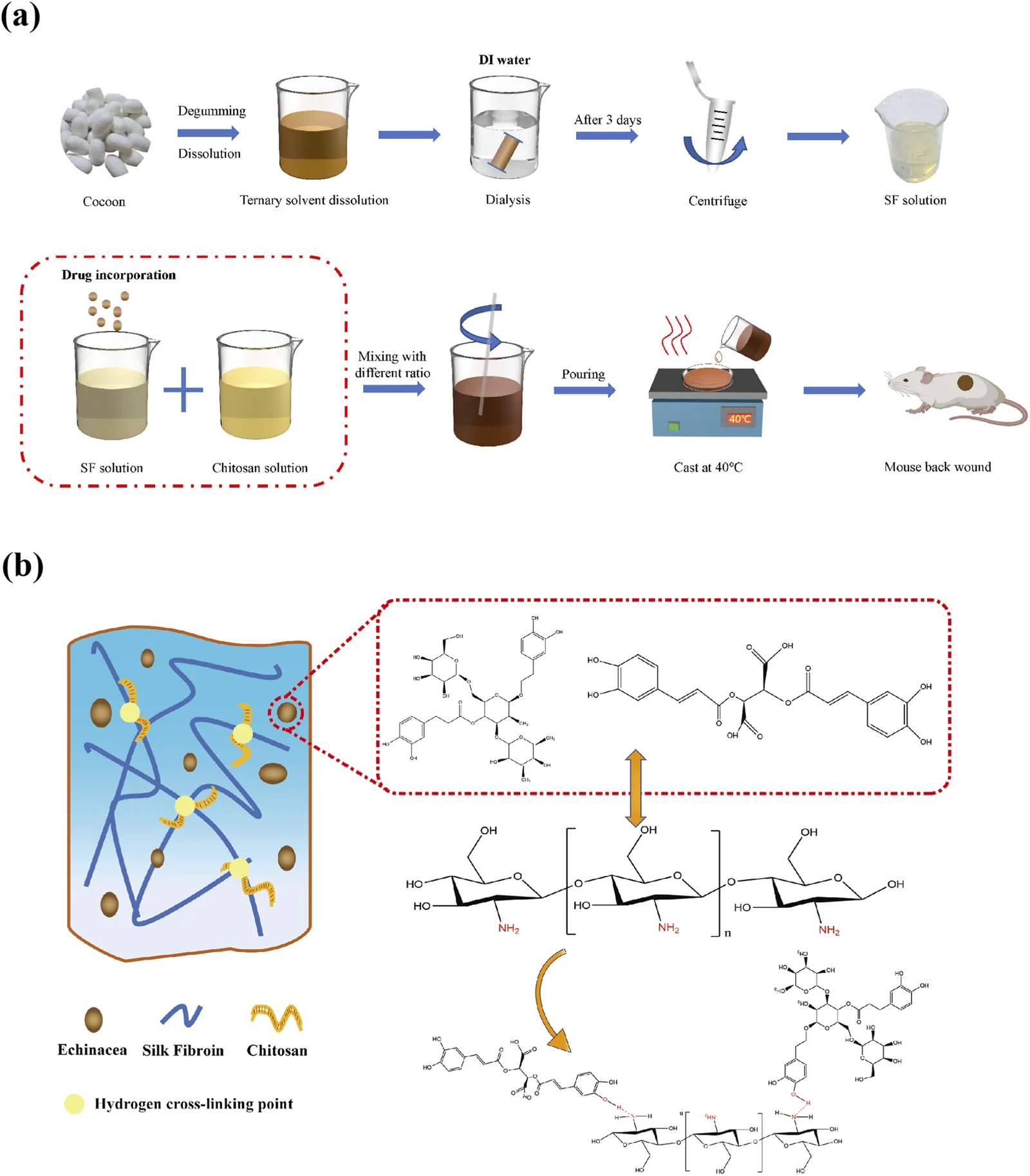
The preparation process and structure diagram of ESC. **(a)** Schematic representation of Echinacea-functionalized silk fibroin (SF)/Chitosan (CS) composite scaffold preparation, **(b)** in-ternal network structure of ESC.

Cocoons were cut into small pieces and boiled in a 2 L solution of 0.2 mol L^−1^ sodium carbonate for 1 h to remove sericin. The SF was washed with deionized water and dried completely in an oven at 50 °C. Next, the dried SF was dissolved in the ter-nary solvent and placed in a water bath at 75 °C (with a mass ratio of CaCl_2_:C_2_H_5_OH:H_2_O = 1:2:8). The obtained solution was dialyzed with a dialysis bag for 3 days. Following this, the SF solution was centrifuged at 9,000 rpm for 15 min, and the resulting supernatant was stored at 4 °C for future use. Figure 1a illustrates the preparation process.

### 2.3 Preparation of Echinacea-functionalized SF/CS transparent scaffolds

Echinacea functionalized SF/CS transparent scaffold was prepared using the pouring method. Firstly, 2 wt% CS solutions and 2 wt% SF solutions were prepared. Then, 30 mg of Echinacea extract was dissolved in the SF solution. Secondly, CS and the above solution were mixed in different ratios of 3:1, 1:1, and 1:3, marked as FC25, FC50, and FC75, respectively. The mixed solution was then ultrasonically dispersed for 20 min. Thirdly, the mixture (5 mL) was poured into the mold and cast at 45 °C until the weight was constant. Finally, the Echinacea functional SF/CS scaffold was obtained after demolding at room temperature.

### 2.4 Characterization

The chemical structure of each component scaffold was identified using attenuated total reflection Fourier transform infrared spectroscopy (FTIR-ATR, Thermo- IS-50, Massachusetts, United States), number of scans 28, resolution 4. The ESC scaffold was cut to a size of 1 cm × 1 cm, and the surface and cross-section morphology of the ESC scaffold were observed using SEM (Zeiss Sigma 300, Oberkochen, Germany) after the vacuum treatment.

### 2.5 Mechanical properties

The universal mechanical testing machine (MTS-E43, Minnesota, United States) was used to conduct tensile strength and adhesion tests. The ultimate tensile test parameter was set at 60 mm min^−1^. The adhesion test used the method of applying the material between two pieces of pig skin, and the test was performed at a speed of 5 mm min^−1^. The adhesion was calculated using the following formula 1:

$$\text{Adhesive }\left(\text{Kpa}\right)=\frac{\mathrm{Max}\;\text{stress}\;\left(N\right)}{\text{Adhesive}\;\text{area}\;\left(m^{2}\right)}\times \frac{1}{1000}$$ (1)

### 2.6 UV light transmittance

A UV spectrophotometer (UV 3600, Shimadzu, Japan) was used to test the transmittance of the ESC scaffold with different ratios in the range of 400–800 nm.

### 2.7 Water vapor transmission test

The permeability of the composite scaffold was tested using the water vapor transmission rate (WVTR) method according to American Society for Testing and Materials (ASTM) E96. ESC scaffold was cut into circular pieces and placed on the top of a glass bottle (inner diameter: 3.5 cm) filled with normal saline. The initial weight of the recording system is W_0_. The glass bottle was then placed in a 37 °C incubator, and the relative humidity was controlled at 50% ± 5%. After 24 h, the total weight of the measurement system is recorded as W_24_, and then the WVTR is calculated according to formula 2.

$$\text{WVTR }\left(gm^{-2}{\text{day}}^{-1}\right)=\frac{W_{0}-W_{24}}{S\times T}\times 100\%$$ (2)

### 2.8 Antimicrobial

#### 2.8.1 Bacterial suspension preparation

Three 12 mL bacterial culture tubes were taken, and 3 mL LB liquid medium was added to each tube. Single colonies were picked from the *Escherichia coli* and *Staphylococcus aureus* solid medium and added to the liquid culture. The other group was used as a blank control. Cells were incubated overnight (15 h) with shaking in a constant temperature oscillator (37 °C, 200 rpm).

#### 2.8.2 Co-culture and plate spread count test

Two samples of each type were cut and placed into the numbered disposable Petri dishes, sterilized by ultraviolet light for 30 min, and then placed in the corresponding 24-well plates. The bacterial solution was diluted to 105 CFU/mL with LB liquid medium, and then the diluted bacterial solution was added to the corresponding numbered bacterial culture tube, which was incubated in a constant temperature oscillator at 37 °C for 24 h.

After the culture was completed, the cells were diluted threefold with sterile PBS solution, and then 100 μL of the co-culture medium was extracted for 10-fold dilutions, and 100 μL of the dilution was uniformly coated on LB solid medium Up. The cells were placed in a constant temperature incubator at 37 °C and cultured for 24 h. Photos were taken, and the number of colonies was recorded.

#### 2.8.3 OD value

A 100 μL sample of the co-culture solution was sucked into a 96-well plate, which was placed on a microplate reader at a wavelength of 600 nm to measure the OD values.

### 2.9 Degradation property test

The degradation behavior of ESC scaffolds in phosphate buffer solution (PBS, PH = 7.4, Macklin, Shanghai, China) was evaluated. The constant weight of the dried freeze-dried scaffold was marked as W0. Then, the samples were immersed in 5 mL PBS and incubated at 37 °C on a shaker. The sample was collected daily and denoted the constant weight as Wt, and the degradation rate was calculated by formula 3. In addition, three sets were repeated for each proportional stent.

$$\text{Degradation ratio }\left(\%\right)=\frac{W_{0}-W_{t}}{W_{0}}\times 100\%$$ (3)

### 2.10 *In vitro* cell assessment for biocompatibility

#### 2.10.1 Live/dead staining

The samples were immersed in 75% alcohol and then disinfected and sterilized under UV light for 2 h NIH3T3 cells were laid on the surface of the material and cultured in sufficient medium after they adhered to the wall. Cellular staining was observed, and photos were taken using a fluorescence microscope (Leica DMi8, Solms, Germany) on the second and third days, respectively.

#### 2.10.2 CCK8

The ESC sample was immersed in alcohol for 20 min and then sterilized with UV for 2 h. After that, ESC was immersed in DMEM for 24 h NIH3T3 cells were cultured in 96 well plates for 12 h. After the cells adhered to the bottom, the cells were washed with PBS, and a DMEM soaking solution was added to keep the culture. CCK8 detection reagent was added at 24, 36, and 48 h, respectively, and the optical density (OD) was measured with an enzyme labeling instrument to evaluate the cell viability. The calculation formula is as follows:

$$\text{Cell viability}=\frac{A_{\text{Drug}\;\text{load}}-A_{\text{Empty}}}{B_{\text{Control}}-A_{\text{Empty}}}\times 100\%$$ (4)

#### 2.10.3 Cell migration

NIH3T3 cells were spread in 6-well plates after being cultured to the third generation. The cells were scratched with equal width in each well when covered with well plates, and the nick spacing of cells was recorded at 0, 24, and 36 h, respectively.

#### 2.10.4 Immunofluorescence

The cells were cultured in a 24-well plate placed with ESC slides for 1 day. The cells were fixed with 4% paraformaldehyde for 10 min and then washed with PBST. Next, 1 mL of 1% Triton X-100 was added to each well at room temperature for 10 min, followed by washing with PBST for 10 min. Then, 5% BSA was added to each well and placed in a CO_2_-free incubator at 37 °C for 1 h. The cellular morphology in ESC soaks was evaluated by rhodamine–phalloidin/DAPI staining.

### 2.11 Wound healing assay of mice

All animal experiments were performed according to protocols approved by the Ethics Committee of Chongqing Medical University (Approval Number: IACUC-CQMU-2022089). Male Kunming mice aged 6–8 weeks were purchased from the Laboratory Animal Center of Chongqing Medical University. All mice were maintained in individually ventilated cages in an IVC animal laboratory with free access to food and water. To evaluate wound healing via a full-thickness skin defect model, the mice were randomly assigned into four groups ((n = 17 per group)): blank group, negative group, positive control group, and experimental group. The negative control group received SF-CS scaffold without echinacea loading, the positive control group received Tegaderm™ Film, and the experimental group received ESC scaffold. The mice were anesthetized by intraperitoneal injection of pentobarbital sodium (50 mg/kg, Sigma-Aldrich), and then the fur was shaved from their backs. A full-thickness excisional skin wound (approximately 8 mm in diameter) was created on the dorsum of each mouse, and the wound area was photographed on days 0, 4, 8, and 12 after treatment (n = 7 mice per group). At the end of the experiment, mice were anesthetized via intraperitoneal injection with pentobarbital sodium (50 mg/kg, 0.3% solution), and then sacrificed by cervical vertebra dislocation. The percent remaining area of wound was calculated by formula 5.

$$\text{Percent remaining area }\left(\%\right)=\frac{S_{\text{day}\;4,8,12}}{S_{0}}\times 100\%$$ (5)

### 2.12 Water contact angle

The appropriate sample size should be taken and placed in the contact angle test platform (Dingsheng JY-82C, Chengde, China). The equipment should then be used in conjunction with the automatic titration system, which will drop water droplets onto the sample. Test photos should be taken, and the goniophotometric method of measuring the contact angle should be employed.

### 2.13 Hemostatic assay test of rat

A total of 9 male SD rats, approximately 6–7 weeks old were randomly divided into three groups (n = 3 per group). The rats were anesthetized with pentobarbital sodium and placed in the supine position, the limbs were fixed, and the liver was exposed by surgery. A pricking needle was used to pierce the liver, followed by the attachment of Tegaderm™ Film and ESC dressing, respectively. The amount of bleeding was used to evaluate the hemostatic ability of the different dressings (Figure 6b).

### 2.14 Histology analysis

On postoperative days 4, 8, and 12 days, the mice were sacrificed (n = 5 per time point). The samples of wound skin and subcutaneous tissue were collected and fixed with paraformaldehyde for 48 h. Tissue samples were embedded in paraffin and sliced into 5-μm sections. Hematoxylineosin (HE) staining, Masson, and immunohistochemical staining were performed after tissue sectioning. Histological and immunohistochemical sections were observed under a light microscope (Leica DM6B, Solms, Germany). IL-6 sections were observed with a confocal microscope (Leica Sp8, Solms, Germany).

### 2.15 Statistical analysis

All data are presented as the mean ± standard deviation (SD) of at least three replicate samples and were analyzed using SPSS 22.0 statistical software (SPSS Inc., Chicago, United States). Student’s t-test was used for comparisons between two groups, while one-way analysis of variance (ANOVA) followed by the *post hoc* test was applied for multiple group comparisons. Repeated-measures ANOVA was used to analyze the longitudinal wound closure data (wound area measured on days 0, 4, 8, and 12). ImageJ (Version 1.53) was used for animal wound and cell image analysis. Origin 2022 was adopted for producing figures. Statistically significant differences are defined as *p < 0.05, **p < 0.01, and ***p < 0.001.

## 3 Results

### 3.1 Preparation and characterization of ESC

**FIGURE 2:**
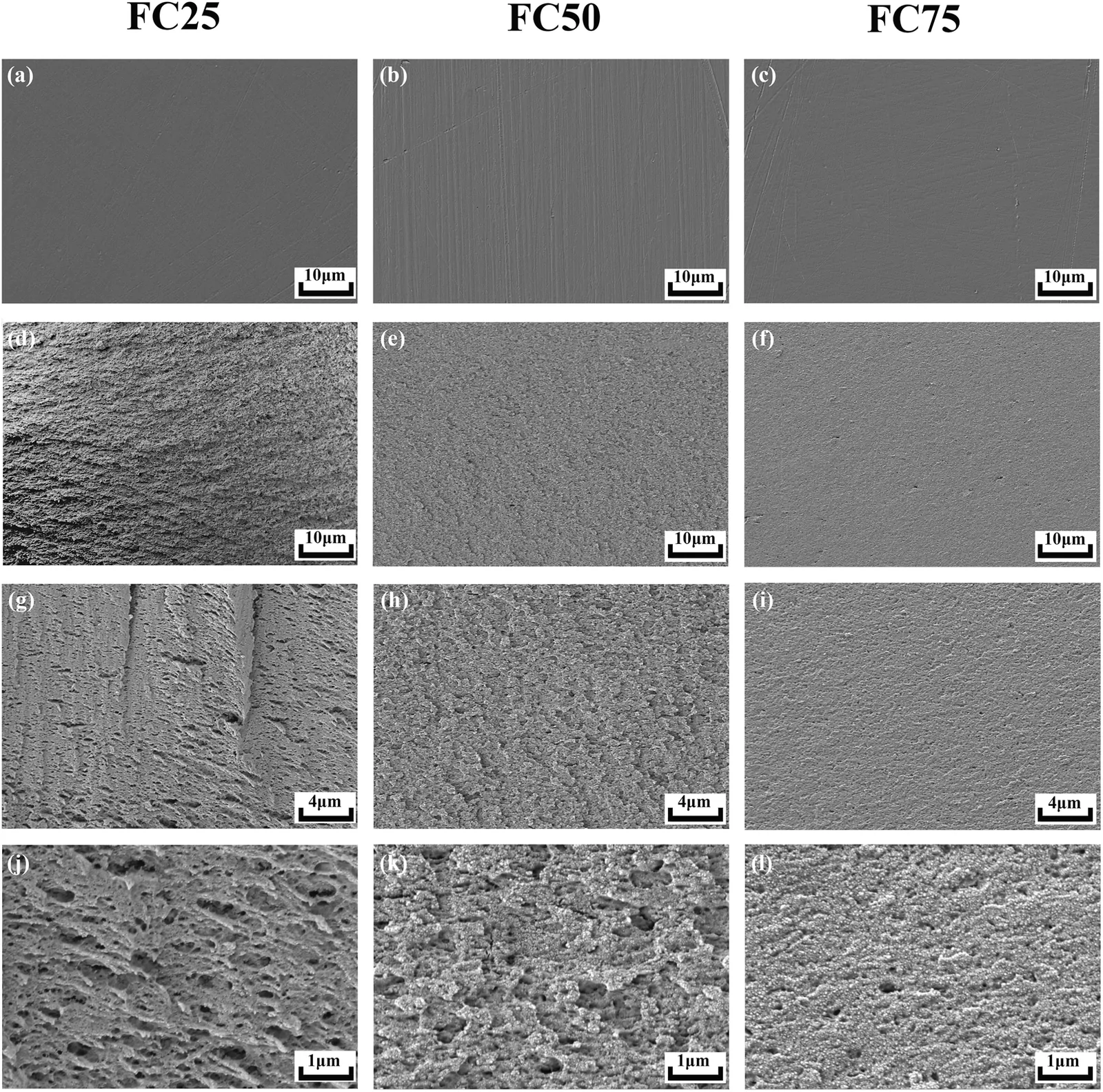
SEM diagram of ESC with three ratios. **(a–c)** Surface, **(d–l)** cross-section.

The Echinacea extract (ESC) was prepared by the casting method, whereby the substances formed chemical bonds with the chitosan molecules (Figure 1), and the results of SEM characterization of the morphology of ESC dressings prepared with different SF-CS ratios are shown in Figure 2. The surface of ESC is smooth, and the structure is affected by the SF doping amount, with irregular fibers visible in both FC25 and FC75. When the SF content reached 50%, the fibers in FC50 were distributed uniformly and consistently. Figure 2d shows cross-sections of ESC films exhibiting a dense mesoporous structure. Increasing the SF content from 25% to 75% increased in-ternal density and fiber stripe visibility but decreased porosity. Together with the results of mechanical analysis, the results of adding different ratios of SF and CS to ESC showed that the SF/CS ratio greatly influenced the internal structure and mechanical properties of ESC. Therefore, the physical properties of ESC can be adjusted by changing the SF/CS ratio.

**FIGURE 3:**
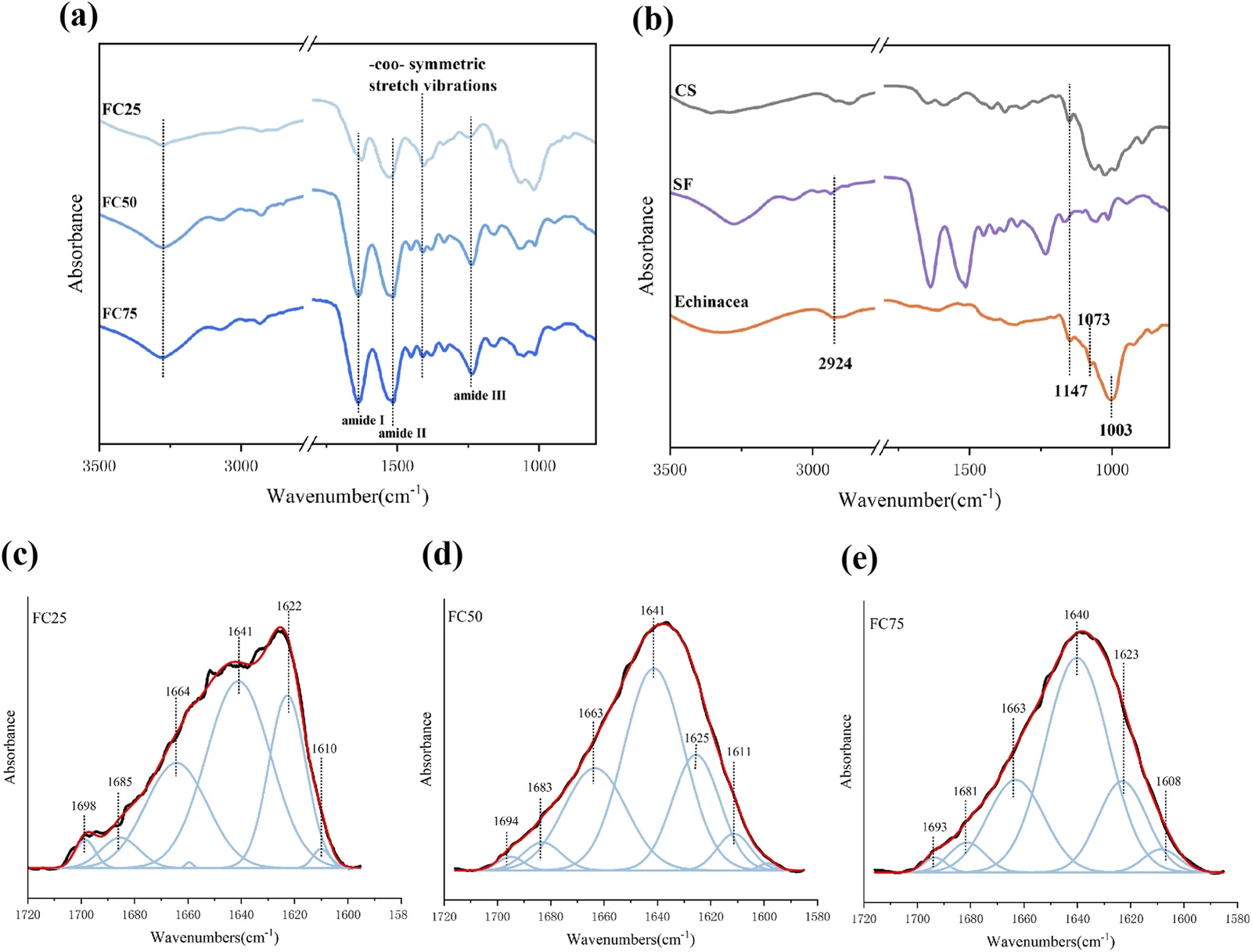
FTIR spectra. **(a)** ESC scaffold with different ratios, **(b)** pure SF, CS and Echinacea, **(c–e)** FTIR deconvolution of amide І band of different ESC scaffold.

Functional groups were characterized and analyzed by FTIR spectroscopy (Figures 3a,b). The characteristic peaks of amide I, amide II, and amide III of ESC prepared with different SF/CS ratios are clearly observed, and the rest of the characteristic peaks are listed in Table 1. In addition, peaks of amides I and II of FC25 shifted with respect to FC50 and FC75, indicating that SF and CS underwent conformational changes. In the CS spectrum, peaks at 1,147 cm^−1^, 1,073 cm^−1^, and 1,055 cm^−1^ corresponded to the stretching vibrations and ring-loading vibrations of C-OH, C-O-C, and C-C in the polysaccharide. Since CS has a positive charge following acidification, symmetric and asymmetric bending vibrational peaks of NH^3+^ appear at 1,635 cm^−1^ and 1,421 cm^−1^, respectively. In the ESC spectrum, peaks near 2,924 cm^−1^ are characteristic of Echinacea leaves, while peaks at 1,234–1,333 cm^−1^ are the stretching and bending resonances of the carboxy-derived carbon–oxygen groups of phenols (Capek et al., 2015). Moreover, arabinogalactan, one of the major polysaccharide components of Echinacea, is responsible for low-intensity bands at 861 cm^*−*1^ (Dobrange et al., 2019).

**TABLE 1:** FTIR peaks of the ESC scaffolds prepared with different SF ratios.

SF/CS blend ratios					Attribution of absorption peak
100/0	25/75	50/50	75/25	0/100	
1,223	1,248	1,237	1,235	-	Amide III
1,409	1,405	1,409	1,409	1,422	-Coo- symmetric stretch vibrations
1,514	1,531	1,515	1,514	-	Amide II
-	1,610	1,613	1,608	-	(Tyr) side chains/aggregated strands
1,637	1,622	1,631	1,623	-	β-Sheets
-	1,641	1,651	1,640	-	Random coils
1,668–1,690	1,664–1,698	1,679–1,694	1,681–1,693	-	Turns
-	1,698	1,694	1,693	-	Antiparallel β-sheet

The proportion of β-sheet content in ESC containing different SF ratios was further examined by deconvolution of the FTIR spectra followed by curve fitting (Figure 3c–e), and the results of quantitative analysis are listed in Table 2. The β-sheet content decreased from 23.74% in FC75% to 17.46% in FC25, while random coils increased from 44.73% to 53.24% due to the acidic environment [7]. The above results demonstrate that the mechanical properties of SF-CS scaffolds can be readily manipulated by varying SF concentration in the composite scaffolds.

**TABLE 2:** The proportion of amide І-associated conformations distributed in SF/CS scaffolds.

Range (cm^−1^)	Assignment	SF	FC25	FC50	FC75
		Percentage (%)			
1,676–1710	Antiparallel β-Sheet	6.03	6.34	5.24	5.57
1,661–1,685	β-Turn	23.34	28.26	27.89	24.73
1,640–1,660	Random coils	66.23	44.73	45.37	53.24
1,616–1,635	β-Sheet	4.37	23.74	20.43	17.46

### 3.2 Physicochemical properties of ESC

#### 3.2.1 Mechanical performance

**FIGURE 4:**
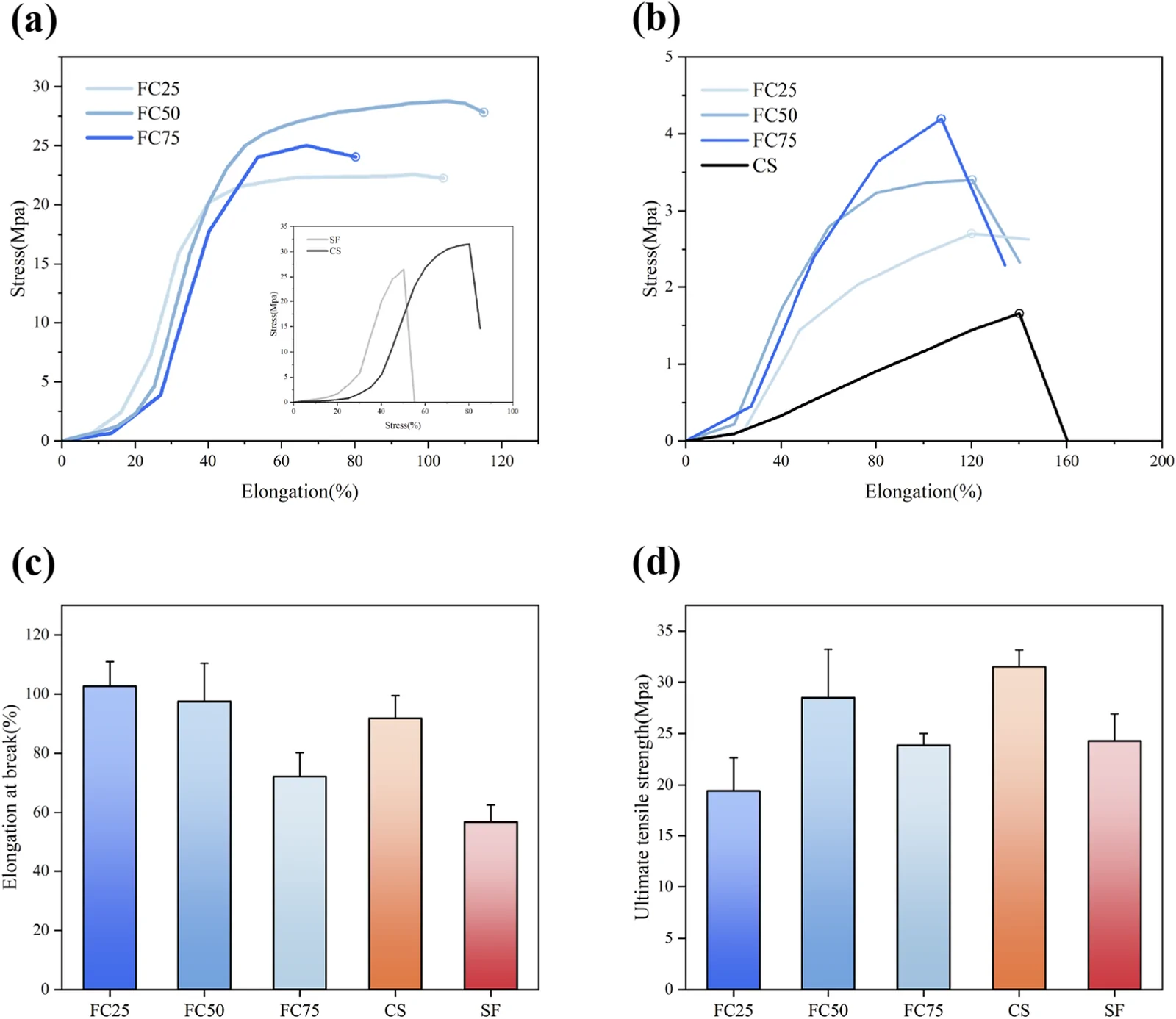
Mechanical properties of ESC scaffolds. **(a)** Tensile stress–strain curves of ESC scaffolds, **(b)** tensile stress–strain curves of ESC scaffolds under wet conditions, **(c)** maximum elongation of ESC scaffolds with three blend ratios at the break, **(d)** ultimate tensile strength value of ESC scaffolds with different ratios, pure SF and CS.

Mechanical properties are key indices for evaluating the performance of wound dressings; ideal dressings have strong tensile strength and deformation resistance to ensure they are not easily broken. The internal structure of the ESC dressing is shown in Figure 4. An interpenetrating network in the system is formed by SF chains and the CS double-helical structure, with Echinacea molecules interspersed in the network. Notably, the amino groups of chitosan form hydrogen bonds with the hydroxyl groups of Echinacea molecules, giving ESC superior mechanical performance.

The mechanical properties of ESC were tested for materials prepared with different ratios and with pure SF/CS in dry and wet states. Figure 4a,b shows the stress–strain curves of composite scaffolds. Increasing the SF content increased the brittleness and length of ESC, and the values for FC25, FC50, and FC75 were 102.6%, 97.5%, and 72.1%, respectively. FC50 performed better than FC25 and FC75 (Figure 4d). In addition, the stress of ESC decreased to 2.7 MPa following immersion in deionized water, indicating that ESC maintains optimal mechanical properties in the wet state and FC50 has a uniform pore distribution and ideal SF fiber anisotropy.

#### 3.2.2 Degradation capacity

**FIGURE 5:**
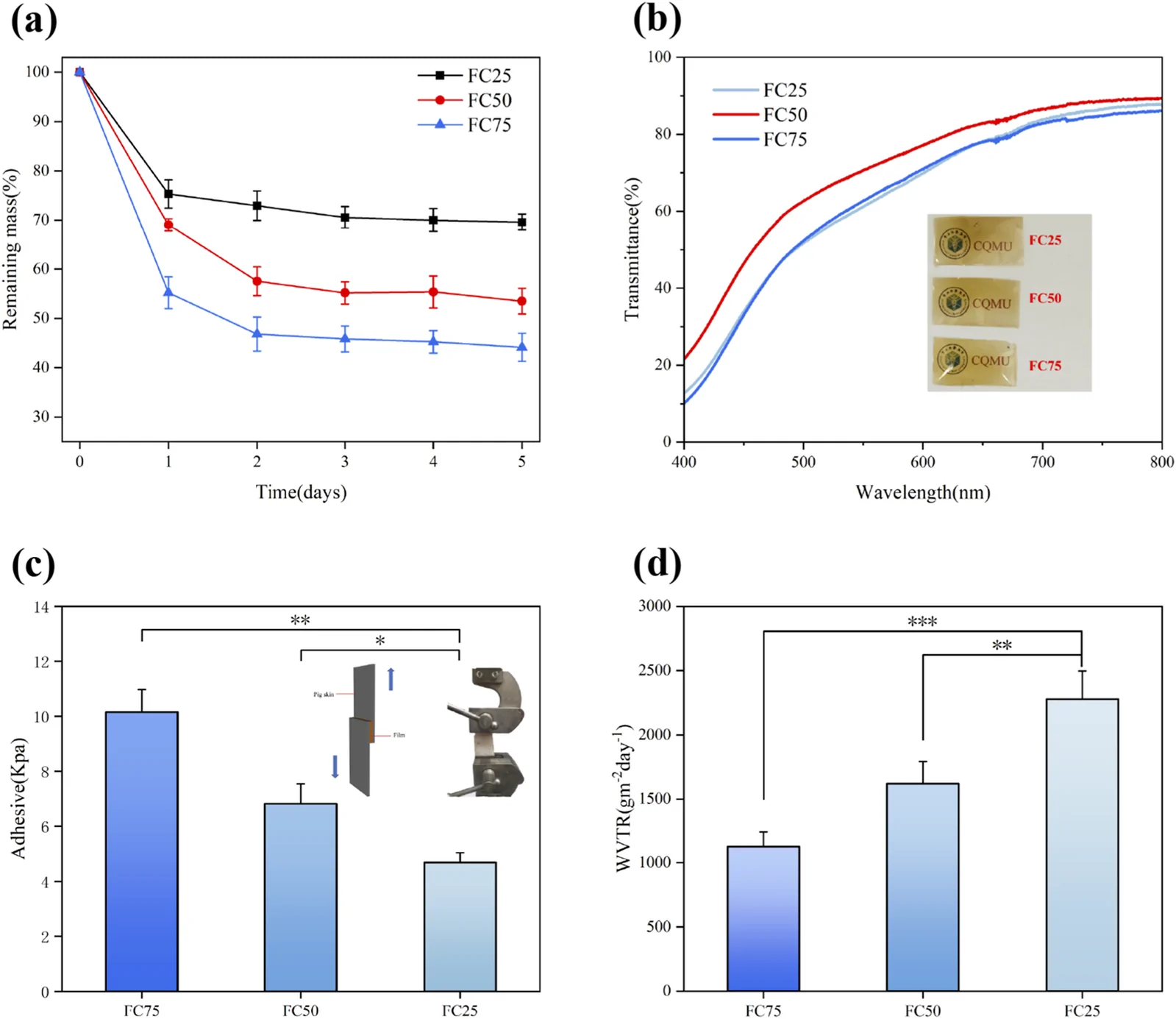
Physical properties experiment of ESC prepared with different ratios. **(a)** Degradability of ESC in PBS, **(b)** transparency of ESC at visible wavelengths, **(c)** adhesive strength of the prepared ESC, **(d)** WVTR of the prepared ESC. The data are presented as the mean ± SD (n = 5). * *p* < 0.05, ** *p* < 0.01, *** *p* < 0.001.

Material degradation enhances the release of drugs and related substances, thereby promoting tissue regrowth. The degradation ability of ESC prepared with different SF ratios in PBS was investigated in this work. As shown in Figure 5a, ESC exhibited good degradation ability, and the degradation rate increased with increasing SF content. FC75 showed a maximum degradation rate of 55% on day 1, which gradually decreased and stabilized after 3 days, demonstrating the release of most of the molecules bound by ESC. This also ensures that proteins, polysaccharides, and Echinacea molecules are released in a timely manner, coordinated with the normal dressing change cycle, thereby contributing to rapid early wound recovery.

#### 3.2.3 Water vapor transmission of ESC

Oxygen plays a crucial role in wound healing, not only by minimizing wound inflammation and infection but also by preventing bacterial infection and promoting wound healing (Yip, 2015). Therefore, the permeability of dressings is a key factor influencing their therapeutic effects. Figure 5d shows that the permeability values of ESC were 1,125 ± 115 (FC75), 1,620 ± 173 (FC50), and 2,278 ± 218 (FC25) g m^−2^ day^−1^. Increasing SF con-tent increases internal density and decreases porosity, resulting in a decrease in WVTR of the ESC composite (Tsao et al., 2010).

#### 3.2.4 Transparency

Visualization of the wound healing process is now possible, and transparent dressings enable real-time observation of wound condition, allowing timely intervention should problems arise. Figure 5b shows that ESC dressings fabricated using SF-CS displayed good transparency, allowing colors to be differentiated through the dressing. In addition, ESC dressings prepared using different SF ratios showed better transmittance in the visible range. FC50 reached more than 80% transmittance in the 620–800 nm range and nearly 90% at shorter wavelengths.

#### 3.2.5 Adhesive properties

Adhesion of trauma material allows the dressing to be tightly bonded to the wound for drug release, hemostasis, and treatment. Figure 5c shows that the adhesive strength of ESC prepared with different SF ratios to pig skin varied considerably. The adhesive strength was highest (10.15 kPa) when the SF content was 75%. The strong adhesion properties were attributed to the synergistic effect of hydrogen bonding, covalent Schiff base formation, and hydrophilic interaction between ESC and pig skin. Another crucial factor contributing to the higher adhesion of FC75 relative to that of FC25 was the enhanced adhesion conferred by the dissolution of SF. Moreover, ESC adhered more firmly to the wound than traditional dressings without relying on adhesive.

#### 3.2.6 Hydrophilic angle

**FIGURE 6:**
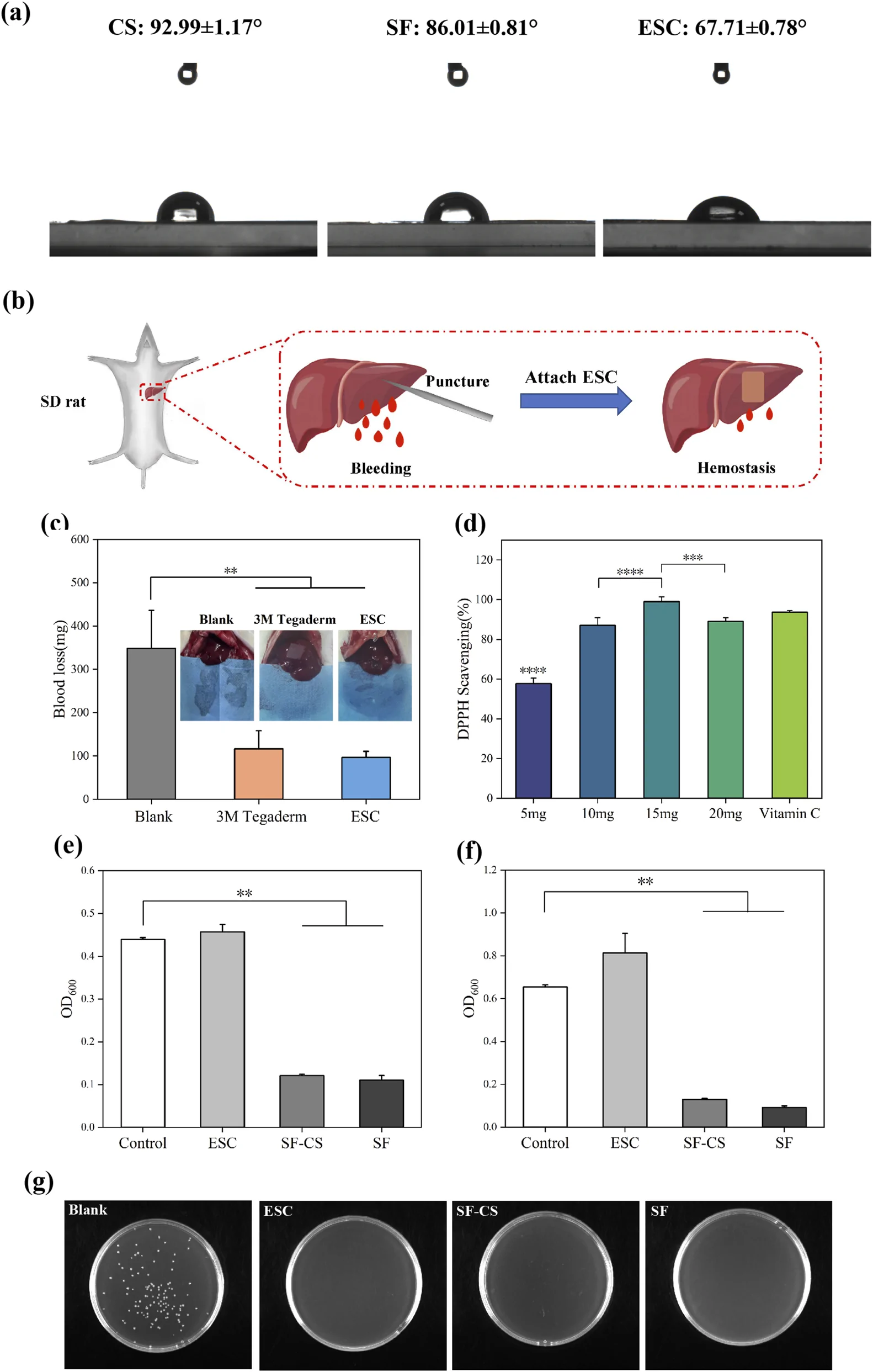
Physical characteristics and *in vitro* antibacterial ability of ESC. **(a)** Water contact angle of ESC, pure CS and SF, **(b)** schematic diagram of ESC hemostatic ability test, **(c)** hemostatic capacity, **(d)** DPPH free radical scavenging ability of Echinacea; bacteriostatic properties of different groups of materials after co-culture with bacteria: **(e)** *Escherichia coli*, **(f)** *Staphylococcus aureus*, **(g)** the number of bacteria in different groups of materials after 24 h incubation on AGAR plates. Data are presented as the mean ± SD (n = 5). ** *p* < 0.01, *** *p* < 0.001, **** *p* < 0.0001.

Abundant functional groups endow the film with strong hydrophilicity, as shown in Figure 6a. The water contact angles of CS and SF were 92.99° ± 1.17° and 86.01° ± 0.81°, respectively. Furthermore, ESC showed significant hydrophilicity with a hydrophilic angle of 67.71° ± 0.78° due to the promotion of β-fold formation by ultrasound and heating during the preparation of ESC, which enhanced the hydrophilicity of SF. This property endows ESC with the ability to absorb wound tissue exudates and reduce wound infection.

### 3.3 *In vivo* hemostatic evaluation and DPPH elimination of ESC

Dressing hemostatic performance was evaluated using a liver hemorrhage model in SD rats, and the results are shown in Figure 6c. The bleeding volume of ESC was 94.7 ± 14.0 mg, and the hemostatic performance was better than that of Tegaderm™ Film (116.6 ± 41.4 mg). This strong hemostatic ability is due to the polysaccharide components of CS and the hydrophilicity of SF, endowing ESC with excellent *in vivo* hemostatic capacity and overcoming the challenges of rapid wound hemostasis.

Polyphenols and polysaccharides in Echinacea can act as effective scavengers of free radicals and play a key role in antioxidative damage. As shown in Figure 6d, when the concentration of Echinacea was 5 mg/mL, the DPPH scavenging rate was low and significantly different from that of other groups (p < 0.0001). The scavenging ability of Echinacea was the same as that of vitamin C (positive control) when the concentration was >10 mg/mL. In addition, the strongest antioxidant capacity and the highest DPPH clearance rate (99.03%) were observed when the concentration of Echinacea was 15 mg/mL.

### 3.4 Antibacterial

Both SF and SF-CS membranes exhibited antibacterial activity against *E. coli* and *S. aureus*, and the antibacterial activity of SF was due to the presence of Ca^2+^ in the ternary solvent. Figures 6e–g shows that SF-CS exhibits excellent antimicrobial activity because CS binds to negatively charged substances in bacteria and negatively affects their functions, resulting in bacterial death (Cheng et al., 2024; Gupta et al., 2022). Furthermore, ESC had a superior synergistic antibacterial effect, and the antibacterial rate reached 100%. Therefore, ESC has excellent antibacterial activity. The ESC scaffold balances gas exchange and microbial barrier through a dual mechanism. Its mesoporous structure (pore size <1 μm) is smaller than that of common wound pathogens (such as, *E. coli* and *S. aureus*), thereby physically blocking bacterial invasion.

### 3.5 Biocompatibility test

#### 3.5.1 CCK8

**FIGURE 7:**
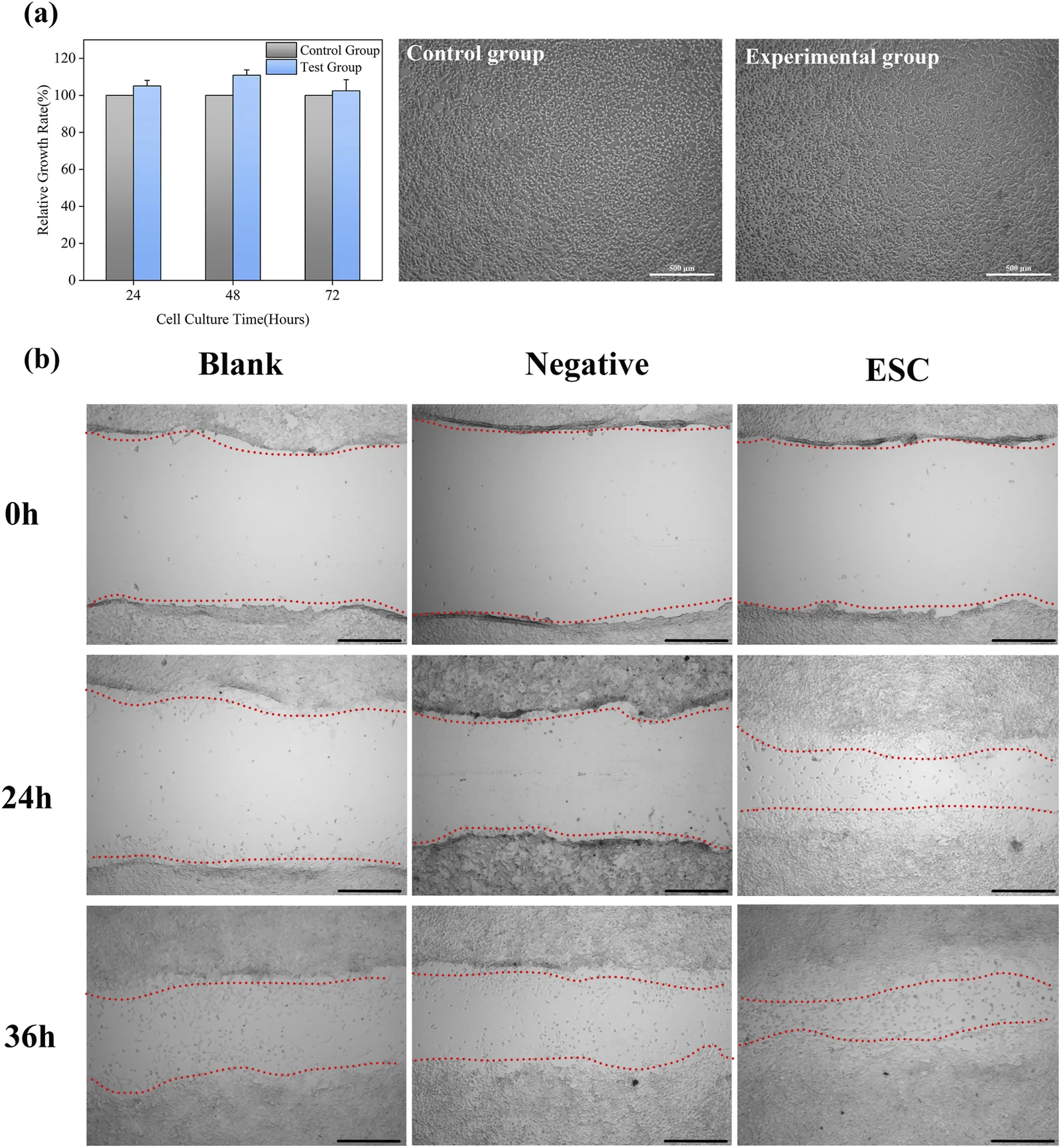
*In vitro* biocompatibility test of ESC. **(a)** The 72 h cell proliferation rate and cell morphology of the control group and ESC treatment group at 48 h. **(b)** Cell migration of blank, ESC, and SF-CS (negative group) treatment. Scale bar: 500 μm. Data are presented as the mean ± SD. All the experiments were independently conducted three times. ** *p* < 0.01, *** *p* < 0.001, **** *p* < 0.0001.

Cell proliferation and cytotoxicity are critical factors for accelerating skin wound healing. Figure 7a shows the cell proliferation rates of NIH3T3 fibroblasts cultured for 24, 48, and 72 h, respectively. Cells grew normally, and the cell proliferation rate peaked at around 48 h, then decreased. The cell proliferation rate in the experimental group was not affected (Figure 7a), indicating that ESC did not negatively affect cell growth. In addition, cell morphology observation under a microscope revealed no significant differences between the two groups, suggesting that ESC has excellent cell biocompatibility and no cytotoxicity.

#### 3.5.2 Cell migration and antibacterial activity

Cell migration is critical in the wound-healing process; hence, we explored the effects of ESC and SF-CS on cell migration. Figure 7b shows that ESC membranes stimulated NIH3T3 cell migration compared with SF-CS and blank control groups after incubation for 36 h, indicating that substances released from ESC promoted cell migration more than those released in the other two groups.

#### 3.5.3 Dead and living cells were stained

**FIGURE 8:**
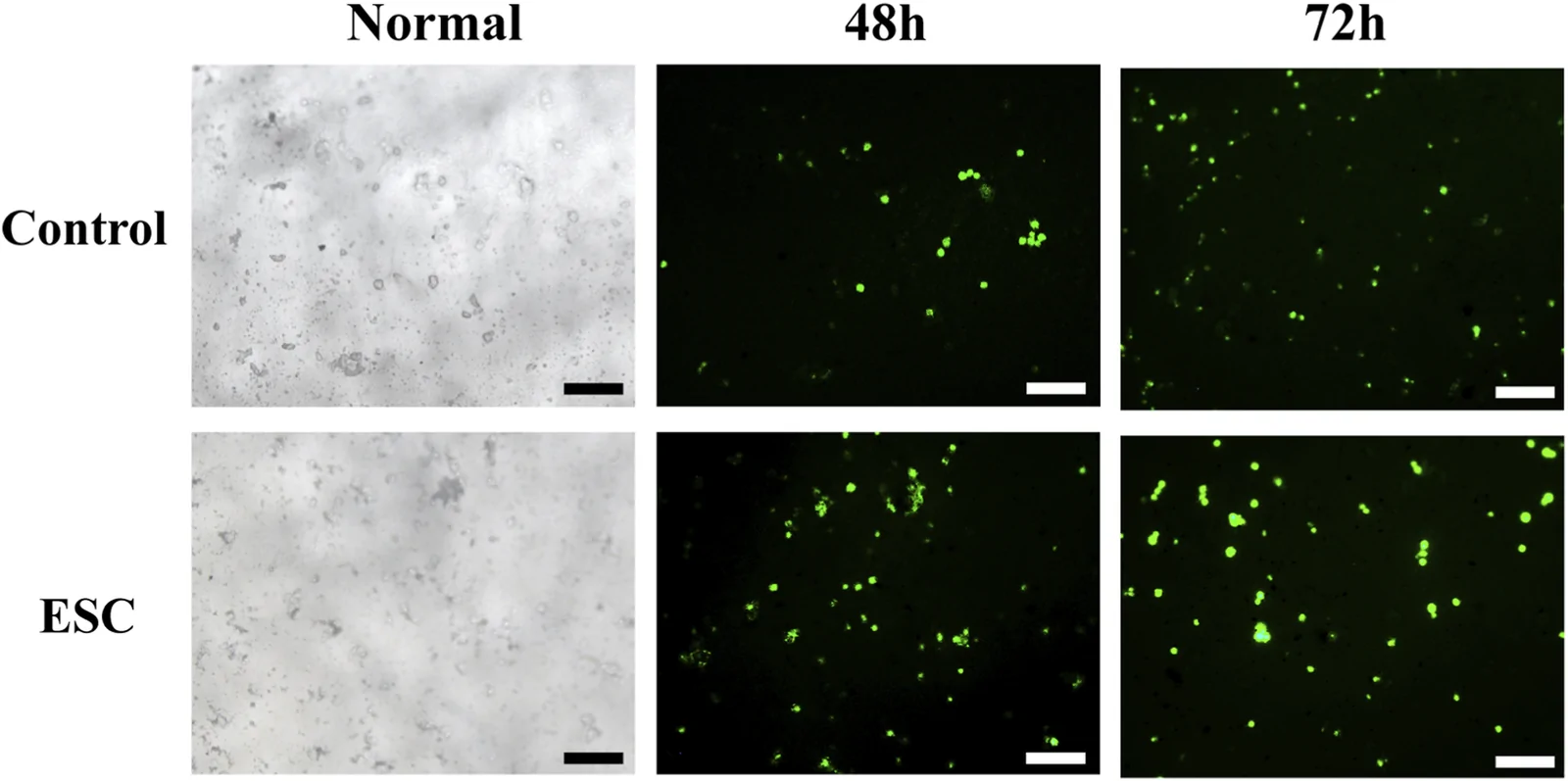
*In vitro* biocompatibility test: NIH3T3 cells co-culture with ESC and SF-CS scaffold for 72 h. Data are presented as the mean ± SD. All the experiments were independently conducted three times. Scale bar: 100 μm.

The cytocompatibility of ESC was further evaluated by dead or alive cell staining. NIH3T3 cells were loaded onto ESC and SF-CS dressings and cultured for 3 days. Results after 48 and 72 h of culture are shown in Figure 8. As culture time was increased, more cells were present in the ESC group than in the SF-CS group, and steady growth continued for up to 3 days without massive cell death.

#### 3.5.4 Immunofluorescence

**FIGURE 9:**
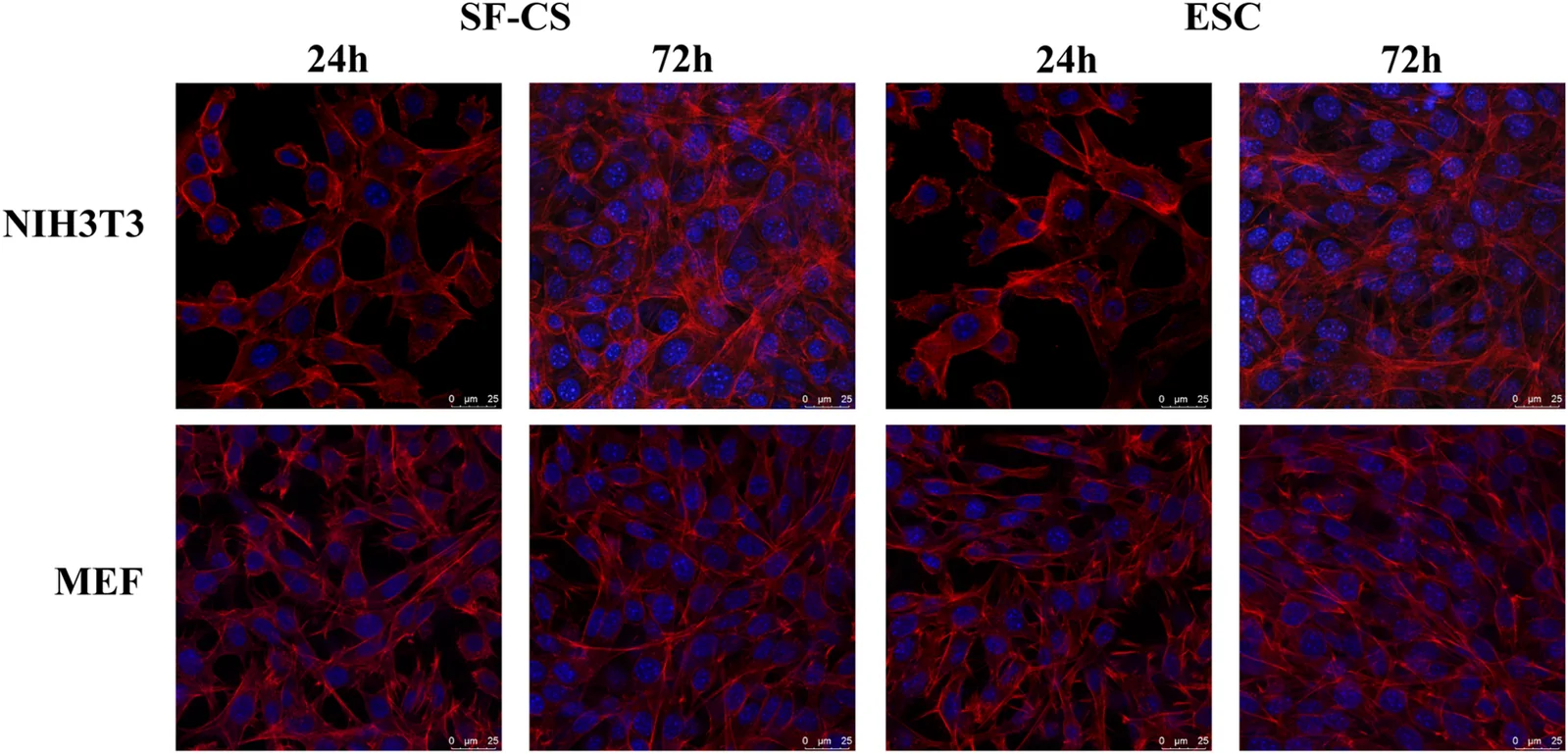
Laser confocal images of NIH3T3 and MEF treated with ESC and SF-CS scaffold for 24 h and 72 h. All the experiments were independently conducted three times. Scale bar: 25 μm.

The effects of ESC on cytoskeletal structure were assessed by rhodamine–phalloidin staining. As shown in Figure 9, ESC-treated NIH3T3 MEF cells grew well with normal cytoskeletal structure, and cell morphology was unchanged, indicating that cell growth was unaffected. The above results collectively proved that ESC has excellent biocompatibility.

### 3.6 *In vivo* wound healing in a full-thickness skin defect model

**FIGURE 10:**
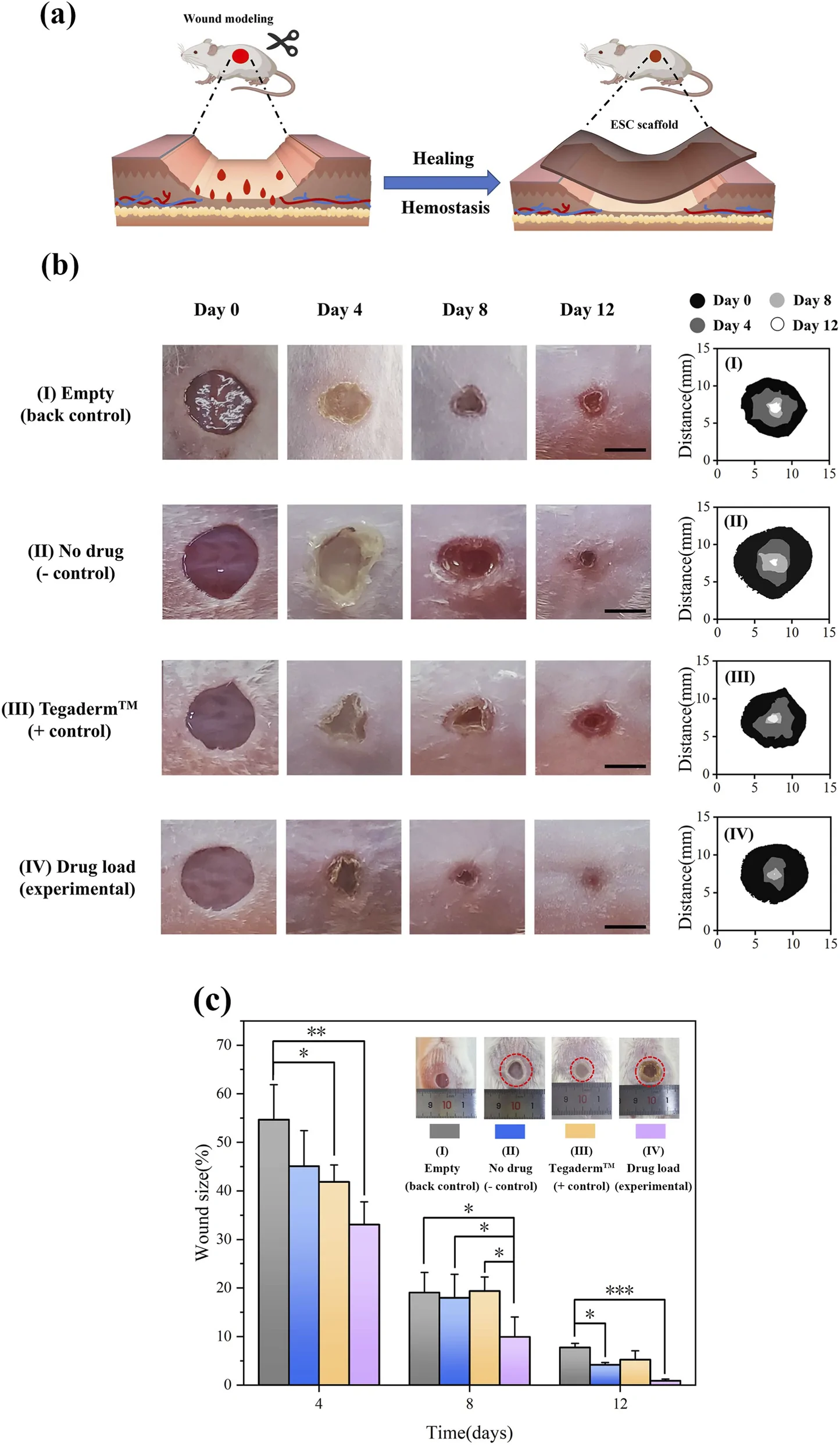
Wound closure progress. **(a)** Schematic of animal experiments, **(b)** representative images of cutaneous wounds treated with ESC, SF-CS, and Tegaderm™ Film at day 0, 4, 8, and 12, **(c)** quantitative analysis of remaining wound area for all groups. n = 7 per group. Data are presented as the mean ± SD. * p < 0.05, ** p < 0.01, *** p < 0.001. Scale bar: 5 mm.

The mouse total cortical excision model was established to evaluate the influence of ESC on skin wound healing (Figure 10a). Blank, SF-CS (negative control), commercial Tegaderm™ Film (positive control), and ESC groups were set up. Skin wounds were photographed, and dressings were changed on days 0, 4, 8, and 12 (Figure 10b). Subsequently, the healing efficiency of each group was calculated from the remaining wound area. The wound size of the ESC group was significantly de-creased, and there was a statistically significant difference between the wound area of the ESC group compared with the 3M and blank groups on day 4 (p < 0.01). It is likely that ESC established cooperative cell–biomaterial niches for wounds, thereby promoting wound healing. On day 8, the ESC group showed impressive healing efficiency; wound healing reached the canter, and the wound and the remaining wound area were significantly smaller than for other groups (p < 0.05). By day 12, the remaining wound area of the ESC group was only 0.86% ± 0.40% and almost completely healed, but in other groups, the wound area was still clearly observed. Furthermore, the healing efficiency of negative and positive control groups was decreased to an extent that was statistically different from the blank group (p < 0.001). The ESC group had the strongest healing effect, followed by the negative control group (Figure 10c).

Echinacea exhibits promising wound healing ability partly due to its polysaccharides, which promote fibroblast growth and stimulate wound healing via a hyaluronic acid–polysaccharide complex that inhibits the production of hyaluronidase. In addition, Echinacea inhibits bacteria commonly found in wounds, such as *E. coli*, *S. aureus*, *Bacillus subtilis*, and *Pseudomonas aeruginosa*, because Echinacoside inhibits hyaluronidase activity. The inhibition of hyaluronidase activity is known to prevent wound infection and reduce wound inflammation (Speroni et al., 2002). Furthermore, according to previous studies, aureole compounds in Echinacea, such as polysaccharides, oxalic acid derivatives, phenols, and amides, activate immunomodulatory pathways by inducing transcriptional changes. On the other hand, the excellent physical properties enable ESC to be efficiently applied to the surface of wounds, thereby isolating it from the external environment while effectively ensuring oxygen reaches the wound. For these reasons, wound healing was promoted more in the ESC group than in the other groups.

### 3.7 Assessment of histological outcomes

**FIGURE 11:**
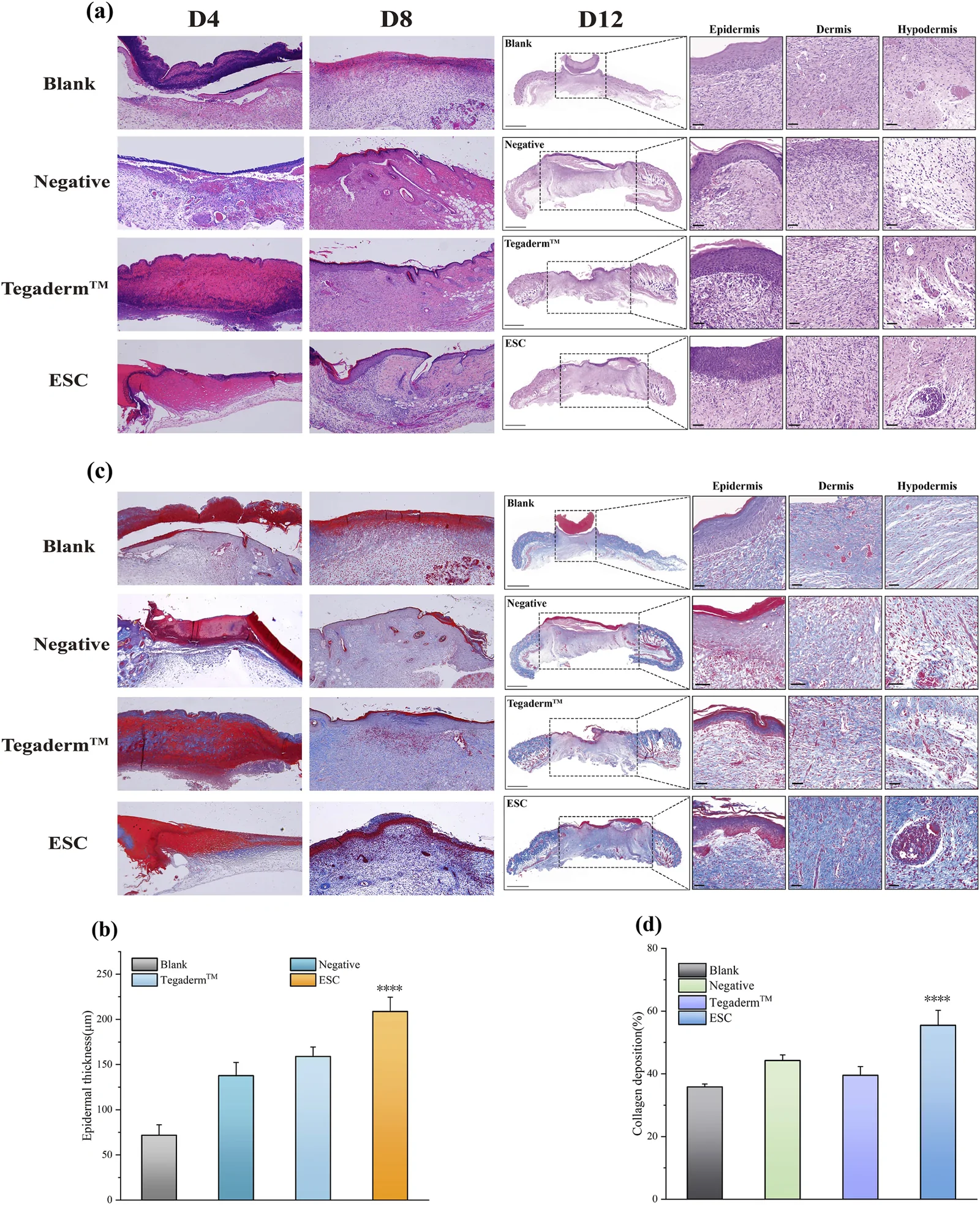
Histopathological analysis of mice wounds by different staining techniques. **(a)** Images of H&E staining on days 4, 8, and 12, **(b)** quantitative analysis of the epidermal thickness in all groups at day 8, **(c)** representative Masson’s staining of skin tissues on day 4, 8, and 12, **(d)** collagen deposition on the 12th day. Data are presented as the mean ± SD. n = 5 per time point. **** p < 0.0001. Scale bar: 200 μm.

In the initial stages of wound repair, the efficiency of wound healing was greatly affected by inflammatory cells. On day 4, granulation tissue and inflammatory cells were observed in all groups to varying degrees. Compared with the blank group, inflammatory cell infiltration was milder in the Echinacea experimental group, which benefited from the anti-inflammatory effect of substances such as chicoric acid and caffeic acid. In addition, in the presence of proteins as well as polysaccharide sub-stances in SF-CS scaffolds, a newly formed epidermis layer was evident, along with numerous blood vessels at the junction between the regenerated tissue and the original tissue, in SF-CS and ESC groups. On day 8, all groups exhibited infiltration of inflammatory cells and a reduction in granulation tissue; the wound area of SF-CS and ESC groups was significantly smaller than on day 4, and a newly formed epidermis was observed in all groups. Furthermore, the ESC group showed the lowest inflammation and capillary regeneration, and the thickness of epithelialization was significantly different from the other groups (Figures 11b,d). On day 12, wounds had almost healed in all groups, but epithelialization and hair follicle formation differed significantly (Figure 11a); dense epithelial tissue and skin appendage formation were observed in 3M and ESC groups, while the skin-healing stage in the SF-CS group occurred later than in the ESC group. Notably, under the influence of Echinacea, hair follicle formation was only observed in the ESC group, indicating that Echinacea could promote hair follicle and epithelium formation.

Collagen expression is one of the key indicators determining the wound healing process. On day 4, collagen deposition was low in all groups but slightly higher in the ESC group (Figure 11c). On day 8, collagen deposition was significantly increased in the ESC and 3M positive control groups compared with blank and negative control groups, indicating that the Tegaderm™ Film and ESC facilitated collagen formation. On day 12, the highest collagen staining intensity was observed in the ESC group, showing significant differences. Compared with the other groups, the epidermal layer in the ESC group was almost completely regenerated, and the dermis was more mature, with neovascularization, mature skin appendages, and hair follicles observed. This indicates that ESC loaded with Echinacea was better able to stimulate collagen deposition, resulting in follicular tissue formation in healed wounds.

**FIGURE 12:**
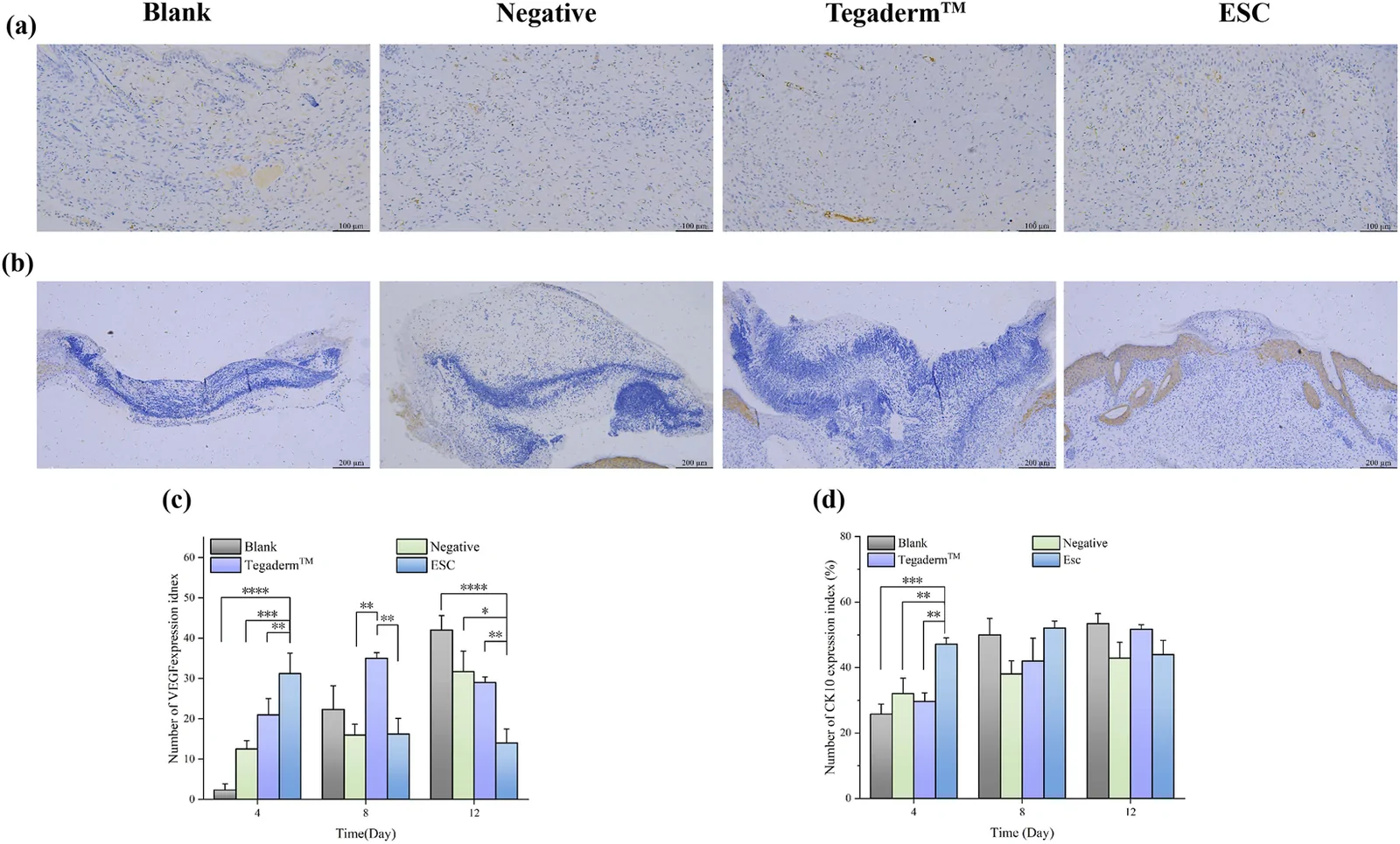
Histopathological analysis of wounds in mice by different immunohistochemical methods. **(a)** VEGF immunohistochemical staining, **(b)** CK10 immunohistochemical staining, **(c)** quantitative analysis: VEGF expression. Black arrow: VEGF positive expression, **(d)** quantitative analysis: CK10 expression. Black arrow: CK10 positive expression. Data are presented as the mean ± SD. n = 5 per time point. ** p < 0.01, *** p < 0.001. Scale bar: 200 μm.

VEGF is one of the most potent crude vascular endothelial cell mitogens in the growth factor family; it promotes vascular endothelial cell growth *in vitro* and induces angiogenesis. Endothelial cell proliferation can be similarly induced under hypoxic wound conditions. In addition, exogenous VEGF promotes angiogenesis, resulting in the enhancement of the early stages of wound healing. Therefore, the decrease in the number of ESC vessels on day 12 occurred after the wound-healing process had entered the tissue remodeling stage and decreased the cell density in the trabecular area. By contrast, new blood vessels with a larger diameter were observed in both blank and 3M groups (Figure 12a). As illustrated in Figure 12c, under the influence of Echinacea, VEGF in the ESC group reached a near-peak level at D 4 and exhibited a near-minimum expression level at D 12, coinciding with the completion of trabecular remodeling. In contrast, the peak VEGF levels observed in the remaining groups occurred at a later stage than that observed in the ESC group, indicating that ESC pro-moted the healing stage and enhanced the early expression of VEGF, thereby accelerating the growth of blood vessels and, ultimately, wound healing.

Cytokeratins (CKs) are keratin proteins located in the epithelial tissue and cyto-plasm that are differentially expressed at different stages of cell differentiation within the epithelial tissue. They can provide scaffolding for epithelial cells and tissues, thus maintaining their structural integrity. The expression levels of CK10 on day 12 are shown in Figure 12b; a keratinized layer was observed in all groups and gradually reached the center of the wound. In Figure 12d, the peak of CK10 expression in the ESC group occurred earlier than in the other groups, which means their keratinocytes exhibited accelerated growth and facilitated wound closure. This indicates that ESC can effectively stimulate the formation of stratum corneum and accelerate the growth of keratinocytes. Therefore, ESC accelerated the expression of CK10 during wound heal-ing and thereby promoted epithelial tissue repair.

### 3.8 Expression of Interleukin-6 (IL-6) during wound healing

**FIGURE 13:**
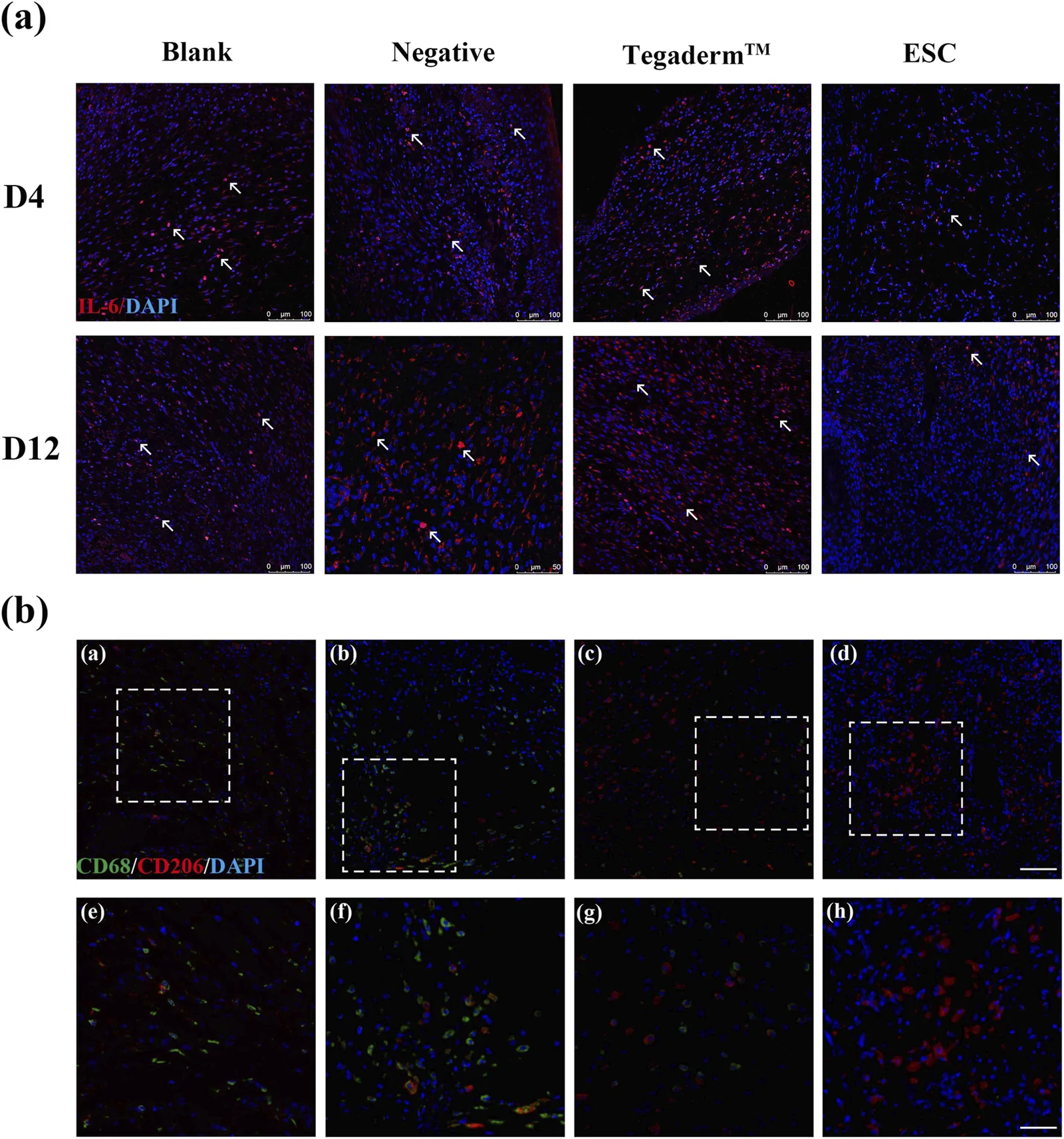
The wounds of mice were stained by the immunofluorescence method with different antibodies. **(a)** Expression of interleukin 6 (IL-6) in each group on days 4 and 12. Scale bar: 100 μm. **(b)** Representative fluorescent images of macrophages at wounds treated with different groups on day 12. Macrophages were marked with pan-maker CD68 (green), and the M2 phenotype was marked with marker CD206 (red). The nuclei were marked with DAPI (blue). Scale bars in (a–d) represent 100 μm, and scale bars in (e–h) represent 40 μm.

To further elucidate the mechanism by which ESC promotes wound recovery, expression of IL-6 was measured in each group on days 4 and 12 to reflect the early and late stages of skin wound healing. As shown in Figure 13, all four groups displayed low IL-6 expression on day 4, which was lowest in the ESC group, indicating different degrees of inflammatory response. On the 12th day, a large amount of IL-6 expression coupled with a high inflammatory response was observed in blank, negative control, and 3M groups, whereas expression remained low in the ESC group. This was at-tributed to the anti-inflammatory and antibacterial effects of Echinacea decreasing the inflammatory responses of tissues. Macrophage phenotypic transition is crucial in the immunomodulation and promotion of scarless healing.

As shown in Figure 13b, only a few macrophages in the blank group reached the M2 stage, while the number was intermediate in the SF-CS group and higher in the 3M group. When Echinacea was introduced into the SF-CS scaffold, ESC significantly accelerated the transformation of macrophages from M1 to M2 compared with other groups. These results were consistent with the degree of inflammation and wound healing.

## 4 Discussion

The evaluation of wound dressings should consider the resistance to bacterial infection and the promotion of scarless wound healing during the healing process of traumatic skin injuries (Deng et al., 2024). Wound dressings prepared with natural materials have attracted much attention in recent years due to their richer functional properties and lower side effects than those using synthetic drugs. Echinacea, a natural plant, contains a variety of natural substances that exhibit many functional properties. Consequently, Echinacea has considerable potential for the current research of multifunctional wound dressings. However, the use of Echinacea as a wound dressing has not been adequately investigated, and its specific mechanism for promoting wound healing has not been fully elucidated.

Cocoons were prepared as a silk protein solution, which was subsequently fused with chitosan and Echinacea extract to create a transparent film dressing. The silk proteins prepared by the ternary solvent method exhibited a high molecular weight and retained part of the fibrous structure, which resulted in a more stable internal structure during the preparation of the dressing and enhanced the deformation resistance of the SF-CS scaffold. This ensured that the dressing was resistant to rupture in different wet and dry wound conditions and had the ability to adhere to the wound surface without the use of an additional adhesive to stop acute hemorrhage. Further-more, the SF-CS stent exhibits a dense pore size in the cross-section, which provides the dressing with a certain degree of breathability and allows it to meet the needs of oxy-gen exchange during the wound healing process.

The future development of multifunctional wound dressings will likely focus on anti-inflammatory, antibacterial, and follicle regeneration promotion properties. The capacity of Echinacea to significantly facilitate scarless healing of skin wounds was validated through the use of mouse skin wounds. The caffeic acid, chicoric acid, and chlorogenic acid present in the Echinacea extract, in conjunction with the residual Ca2+ present in the SF, conferred anti-inflammatory and antimicrobial effects upon the ESC, which effectively inhibited the value-added of *E. coli* and *S. aureus* in the wounds, and solved the problem of susceptibility to infections in the healing process of skin wounds. Furthermore, caffeic acid in Echinacea acts as an oxidant, effectively scavenging free radicals in wounds and reducing oxidative stress. Furthermore, the polysaccharides and proteins present in ESC provided nutritional support for the wound healing pro-cess, which accelerated the wound healing process in conjunction with the medicinal effects of Echinacea.

Echinacea has been demonstrated to significantly promote the formation of skin appendages, facilitate the regeneration of capillaries, and accelerate the process of epithelialization of the wound surface. Immunohistochemical analyses demonstrated that ESC facilitated the production of VEGF factor and CK10 protein, resulting in an earlier peak time than the other groups. Furthermore, these analyses corroborated the ability of Echinacea to promote the transition of macrophages from M1 to M2 and the emergence of follicular hair follicles in the wounds, as observed in D12. Consequently, Echinacea exerts immunomodulatory effects and contributes to the formation of sub-cutaneous neoplastic tissue during wound healing, thereby stimulating the regeneration of skin hair follicles and thus accelerating scarless wound healing.

In existing research, dressings are predominantly in the form of hydrogel, non-transparent film, and electrostatically spun film. These dressings are typically complex to prepare, and the residual substances resulting from chemical cross-linking may affect biocompatibility when used for an extended period in the wound environment (Galili et al., 2024). Furthermore, the dressing itself is susceptible to breakage, which limits its practical application. Furthermore, dressings are typically prepared by incorporating graphene, metal ions, and other chemical compounds. To enhance the performance of dressings in various aspects, a multitude of substances are often incorporated, and some of these substances may be challenging to be absorbed by the body, thereby in-creasing the uncertainty in clinical applications (Vagena et al., 2024). Consequently, dressings based on natural materials are being subjected to in-depth studies. These substances exhibit natural immunomodulation and anti-inflammatory properties, among others. The combination of these substances with stent material, as well as the preparation of different forms of wound dressings by combining these substances with scaffold materials, has led to the development of a range of wound dressings to meet diverse needs. Furthermore, the cost of preparation, material properties, and therapeutic efficacy are all important factors in the evaluation of wound dressings. In comparison to traditional dressings, the scaffold material exhibits adjustable properties, the capacity to carry different drugs, a rapid release of these drugs, enhanced solubility, and reduced toxicity. These characteristics illustrate the multifunctionality of the SF-CS scaffold.

In the future, the SF-CS scaffold will be subjected to testing to ascertain its slow-release effect. This will help wounds to absorb unused doses of drugs at different stages. In addition, the mechanism of Echinacea’s immune modulation will be studied in depth. This will be combined with other drugs for the purpose of treating special wounds. The results of these studies will provide reliable drug carrier materials and lay the foundation for the development of wound dressings with special properties.

## 5 Conclusion

This study describes a new biomaterial, an Echinacea-loaded SF-CS scaffold, for wound healing and contributes to the growing field of skin tissue engineering for wound healing. This work aimed to harness the biological potential of natural products by incorporating them into biomaterials and exploring their potential as a wound care dressing by assessing their *in vitro* biocompatibility and efficiency in promoting skin wound healing. The dense internal structure of ESC confers superior mechanical properties and WVTR, and the dressing does not break easily under wet conditions. The unique components in Echinacea can promote cell migration and efficient DPPH scavenging, while the strong adhesion ability and hydrophilicity of ESC result in rapid hemostasis. HE and Masson staining of wound tissues indicated that ESC accelerated skin growth by promoting hair follicle regeneration, accelerating re-epithelialization and collagen deposition. CK10 and VEGF immunohistochemical analysis showed that ESC significantly promoted skin keratin and VEGF expression. In addition, IL-6 immunofluorescence demonstrated that Echinacea reduces wound inflammation and promotes wound healing. Moreover, experiments using cells confirmed that ESC is biocompatible and does not compromise cellular integrity or alter cytoskeletal morphology. Therefore, ESC shows potential as an ideal transparent dressing for hemostasis, but it still needs further verification in clinical scarless wound healing.

## Data Availability

The original contributions presented in the study are included in the article/Supplementary Material, further inquiries can be directed to the corresponding authors.

## References

[B1] Barrett B. (2003). Medicinal properties of Echinacea: a critical review. Phytomedicine 10, 66–86. 10.1078/094471103321648692 12622467

[B2] Capek P. Šutovská M. Kocmálová M. Fraňová S. Pawlaczyk I. Gancarz R. (2015). Chemical and pharmacological profiles of Echinacea complex. Int. J. Biol. Macromol. 79, 388–391. 10.1016/j.ijbiomac.2015.05.010 25999016

[B3] Castaño O. Pérez-Amodio S. Navarro-Requena C. Mateos-Timoneda M. Engel E. (2018). Instructive microenvironments in skin wound healing: biomaterials as signal releasing platforms. Adv. Drug Deliv. Rev. 129, 95–117. 10.1016/j.addr.2018.03.012 29627369

[B4] Cheng Q. He Y. Ma L. Lu L. Cai J. Xu Z. (2024). Regenerated silk fibroin coating stable liquid metal nanoparticles enhance photothermal antimicrobial activity of hydrogel for wound infection repair. Int. J. Biol. Macromol. 263, 130373. 10.1016/j.ijbiomac.2024.130373 38395280

[B5] Chouhan D. Mandal B. B. (2020). Silk biomaterials in wound healing and skin regeneration therapeutics: from bench to bedside. Acta Biomater. 103, 24–51. 10.1016/j.actbio.2019.11.050 31805409

[B6] Ciganović P. Jakupović L. Momchev P. Nižić Nodilo L. Hafner A. Zovko Končić M. (2023). Extraction optimization, antioxidant, cosmeceutical and wound healing potential of Echinacea purpurea glycerolic extracts. Molecules 28, 1177. 10.3390/molecules28031177 36770844 PMC9920817

[B7] Deng J. Li J. Yan L. Guo W. Ding X. Ding P. (2024). Accelerated, injectable, self-healing, scarless wound dressings using rGO reinforced dextran/chitosan hydrogels incorporated with PDA-loaded asiaticoside. Int. J. Biol. Macromol. 278, 134424. 10.1016/j.ijbiomac.2024.134424 39111509

[B8] Dobrange E. Peshev D. Loedolff B. Van den Ende W. (2019). Fructans as immunomodulatory and antiviral agents: the case of echinacea. Biomolecules 9, 615. 10.3390/biom9100615 31623122 PMC6843407

[B9] Galili U. Li J. Schaer G. L. (2024). Regeneration in mice of injured skin, heart, and spinal cord by α-Gal nanoparticles recapitulates regeneration in amphibians. Nanomater. (Basel) 14, 730. 10.3390/nano14080730 38668224 PMC11055133

[B10] Gupta S. Prasad P. Roy A. Alam M. M. Ahmed I. Bit A. (2022). Metallic ion-based graphene oxide functionalized silk fibroin-based dressing promotes wound healing via improved bactericidal outcomes and faster re-epithelization. Biomed. Mater 17, 035010. 10.1088/1748-605X/ac64dd 35385833

[B11] Hosami F. Manayi A. Salimi V. Khodakhah F. Nourbakhsh M. Nakstad B. (2021). The pro-apoptosis effects of Echinacea purpurea and Cannabis sativa extracts in human lung cancer cells through caspase-dependent pathway. BMC Complement. Med. Ther. 21, 37. 10.1186/s12906-021-03204-6 33446187 PMC7809807

[B12] Hou L. Wang W. Wang M. K. Song X. S. (2022). Acceleration of healing in full-thickness wound by chitosan-binding bFGF and antimicrobial peptide modification chitosan membrane. Front. Bioeng. Biotechnol. 10, 878588. 10.3389/fbioe.2022.878588 35547167 PMC9081572

[B13] Howling G. I. Dettmar P. W. Goddard P. A. Hampson F. C. Dornish M. Wood E. J. (2001). The effect of chitin and chitosan on the proliferation of human skin fibroblasts and keratinocytes *in vitro* . Biomaterials 22, 2959–2966. 10.1016/s0142-9612(01)00042-4 11575470

[B14] Jayakumar R. Prabaharan M. Sudheesh Kumar P. T. Nair S. V. Tamura H. (2011). Biomaterials based on chitin and chitosan in wound dressing applications. Biotechnol. Adv. 29, 322–337. 10.1016/j.biotechadv.2011.01.005 21262336

[B15] Jia B. Li G. Cao E. Luo J. Zhao X. Huang H. (2023). Recent progress of antibacterial hydrogels in wound dressings. Mater Today Bio 19, 100582. 10.1016/j.mtbio.2023.100582 36896416 PMC9988584

[B16] Jiang L. Li W. Wang Y. Zhang X. Yu D. Yin Y. (2014). Effects of cichoric acid extract from Echinacea purpurea on collagen-induced arthritis in rats. Am. J. Chin. Med. 42, 679–692. 10.1142/s0192415x1450044x 24871659

[B17] Ju H. W. Lee O. J. Lee J. M. Moon B. M. Park H. J. Park Y. R. (2016). Wound healing effect of electrospun silk fibroin nanomatrix in burn-model. Int. J. Biol. Macromol. 85, 29–39. 10.1016/j.ijbiomac.2015.12.055 26718866

[B18] Kang D. Liu Z. Qian C. Huang J. Zhou Y. Mao X. (2021). A three-dimensional bioprinting technique, based on a gelatin/alginate hydrogel, for the tissue engineering of hair follicle reconstruction. Int. J. Biol. Macromol. 21, 01927–01929. 10.1016/j.ijbiomac.2021.09.014 34509522

[B19] Kang D. Liu Z. Qian C. Huang J. Zhou Y. Mao X. (2023). 3D bioprinting of a gelatin-alginate hydrogel for tissue-engineered hair follicle regeneration. Acta Biomater. 165, 19–30. 10.1016/j.actbio.2022.03.011 35288311

[B20] Karsch-Völk M. Barrett B. Kiefer D. Bauer R. Ardjomand-Woelkart K. Linde K. (2014). Echinacea for preventing and treating the common cold. Cochrane Database Syst. Rev. 2014, Cd000530. 10.1002/14651858.CD000530.pub3 24554461 PMC4068831

[B21] Li X. Liu Y. Zhang J. You R. Qu J. Li M. (2017). Functionalized silk fibroin dressing with topical bioactive insulin release for accelerated chronic wound healing. Mater Sci. Eng. C Mater Biol. Appl. 72, 394–404. 10.1016/j.msec.2016.11.085 28024602

[B22] Li T. T. Sun L. Zhong Y. Peng H. K. Ren H. T. Zhang Y. (2022). Silk fibroin/polycaprolactone-polyvinyl alcohol directional moisture transport composite film loaded with antibacterial drug-loading microspheres for wound dressing materials. Int. J. Biol. Macromol. 207, 580–591. 10.1016/j.ijbiomac.2022.02.105 35218809

[B23] Ma L. Sun Y. Cheng Q. Yang Z. Wang J. Xu Z. (2023). Silk protein-mediated biomineralization: from bioinspired strategies and advanced functions to biomedical applications. ACS Appl. Mater Interfaces 15, 33191–33206. 10.1021/acsami.3c04067 37417928

[B24] Martin P. Nunan R. (2015). Cellular and molecular mechanisms of repair in acute and chronic wound healing. Br. J. Dermatol 173, 370–378. 10.1111/bjd.13954 26175283 PMC4671308

[B25] Moeini A. Pedram P. Makvandi P. Malinconico M. Gomez d'Ayala G. (2020). Wound healing and antimicrobial effect of active secondary metabolites in chitosan-based wound dressings: a review. Carbohydr. Polym. 233, 115839. 10.1016/j.carbpol.2020.115839 32059889

[B26] Moghtaderi M. Mirzaie A. Zabet N. Moammeri A. Mansoori-Kermani A. Akbarzadeh I. (2021). Enhanced antibacterial activity of Echinacea angustifolia extract against multidrug-resistant Klebsiella pneumoniae through niosome encapsulation. Nanomater. (Basel) 11, 1573. 10.3390/nano11061573 34203811 PMC8232788

[B27] Soon S. L. Crawford R. I. (2001). Recurrent erythema nodosum associated with echinacea herbal therapy. J. Am. Acad. Dermatol 44, 298–299. 10.1067/mjd.2001.112219 11174391

[B28] Speroni E. Govoni P. Guizzardi S. Renzulli C. Guerra M. C. (2002). Anti-inflammatory and cicatrizing activity of Echinacea pallida nutt. root extract. J. Ethnopharmacol. 79, 265–272. 10.1016/s0378-8741(01)00391-9 11801391

[B29] Tsao C. T. Chang C. H. Lin Y. Y. Wu M. F. Wang J. L. Han J. L. (2010). Antibacterial activity and biocompatibility of a chitosan-gamma-poly(glutamic acid) polyelectrolyte complex hydrogel. Carbohydr. Res. 345, 1774–1780. 10.1016/j.carres.2010.06.002 20598293

[B30] Ueno H. Yamada H. Tanaka I. Kaba N. Matsuura M. Okumura M. (1999). Accelerating effects of chitosan for healing at early phase of experimental open wound in dogs. Biomaterials 20, 1407–1414. 10.1016/s0142-9612(99)00046-0 10454012

[B31] Vagena I. A. Gatou M. A. Theocharous G. Pantelis P. Gazouli M. Pippa N. (2024). Functionalized ZnO-Based nanocomposites for diverse biological applications: current trends and future perspectives. Nanomater. (Basel) 14, 397. 10.3390/nano14050397 38470728 PMC10933906

[B32] Weishaupt R. Bächler A. Feldhaus S. Lang G. Klein P. Schoop R. (2020). Safety and dose-dependent effects of echinacea for the treatment of acute cold episodes in children: a multicenter, randomized, open-label clinical trial. Child. (Basel) 7, 292. 10.3390/children7120292 33333722 PMC7765151

[B33] Xie H. Bai Q. Kong F. Li Y. Zha X. Zhang L. (2022). Allantoin-functionalized silk fibroin/sodium alginate transparent scaffold for cutaneous wound healing. Int. J. Biol. Macromol. 207, 859–872. 10.1016/J.IJBIOMAC.2022.03.147 35358577

[B34] Xu W. Zhu H. Hu B. Cheng Y. Guo Y. Yao W. (2021). Echinacea in hepatopathy: a review of its phytochemistry, pharmacology, and safety. Phytomedicine 87, 153572. 10.1016/j.phymed.2021.153572 34029938

[B35] Yang P. Wang D. Shi Y. Li M. Gao M. Yu T. (2020). Insulin-containing wound dressing promotes diabetic wound healing through stabilizing HIF-1α. Front. Bioeng. Biotechnol. 8, 592833. 10.3389/fbioe.2020.592833 33392167 PMC7775506

[B36] Yip W. L. (2015). Influence of oxygen on wound healing. Int. Wound J. 12, 620–624. 10.1111/iwj.12324 24974913 PMC7950466

[B37] Yu N. Li Y. Wang Y. Xu H. Ye F. Fu Q. (2022). Healing effect of carboxymethyl chitosan-plantamajoside hydrogel on burn wound skin. Burns 48, 902–914. 10.1016/j.burns.2022.01.019 35153110

[B38] Zhang W. Chen L. Chen J. Wang L. Gui X. Ran J. (2017). Silk fibroin biomaterial shows safe and effective wound healing in animal models and a randomized controlled clinical trial. Adv. Healthc. Mater 6. 10.1002/adhm.201700121 28337854

[B39] Zhang Y. Chang M. Bao F. Xing M. Wang E. Xu Q. (2019). Multifunctional Zn doped hollow mesoporous silica/polycaprolactone electrospun membranes with enhanced hair follicle regeneration and antibacterial activity for wound healing. Nanoscale 11, 6315–6333. 10.1039/c8nr09818b 30882821

[B40] Zhao P. Zhang Y. Chen X. Xu C. Guo J. Deng M. (2023). Versatile hydrogel dressing with skin adaptiveness and mild photothermal antibacterial activity for methicillin-resistant staphylococcus aureus-infected dynamic wound healing. Adv. Sci. (Weinh) 10, e2206585. 10.1002/advs.202206585 36776018 PMC10104652

[B41] Zheng X. Ding Z. Cheng W. Lu Q. Kong X. Zhou X. (2020). Microskin-inspired injectable MSC-laden hydrogels for scarless wound healing with hair follicles. Adv. Healthc. Mater 9, e2000041. 10.1002/adhm.202000041 32338466 PMC7473495

